# Electrical somatosensory stimulation followed by motor training of the paretic upper limb in acute stroke: study protocol for a randomized controlled trial

**DOI:** 10.1186/s13063-017-1815-9

**Published:** 2017-02-23

**Authors:** Emma Ghaziani, Christian Couppé, Cecilie Henkel, Volkert Siersma, Mette Søndergaard, Hanne Christensen, S. Peter Magnusson

**Affiliations:** 1 Department of Physical and Occupational Therapy, Bispebjerg Hospital, Bispebjerg Bakke 23, bldg. 10, 2400, Copenhagen, Denmark; 2 Faculty of Health and Medical Sciences, University of Copenhagen, Copenhagen, Denmark; 3 Institute of Sports Medicine, Department of Orthopaedic Surgery M, Bispebjerg Hospital, Copenhagen, Denmark; 4 Center for Healthy Aging, Faculty of Health and Medical Sciences, University of Copenhagen, Copenhagen, Denmark; 5 The Research Unit for General Practice and Section of General Practice, Department of Public Health, University of Copenhagen, Copenhagen, Denmark; 6 Department of Neurology, Bispebjerg Hospital, Copenhagen, Denmark

**Keywords:** Acute stroke, Upper extremity, Recovery of function, Electrical stimulation, Rehabilitation, Longitudinal studies

## Abstract

**Background:**

Upper limb paresis is one of the most frequent and persistent impairments following stroke. Only 12–34% of stroke patients achieve full recovery of upper limb functioning, which seems to be required to habitually use the affected arm in daily tasks. Although the recovery of upper limb functioning is most pronounced during the first 4 weeks post stroke, there are few studies investigating the effect of rehabilitation during this critical time window. The purpose of this trial is to determine the effect of electrical somatosensory stimulation (ESS) initiated in the acute stroke phase on the recovery of upper limb functioning in a nonselected sample of stroke patients.

**Methods/design:**

A sample of 102 patients with upper limb paresis of varying degrees of severity is assigned to either the intervention or the control group using stratified random sampling. The intervention group receives ESS plus usual rehabilitation and the control group receives sham ESS plus usual rehabilitation. The intervention is applied as 1 h of ESS/sham ESS daily, followed by motor training of the affected upper limb. The ESS/sham ESS treatment is initiated within 7 days from stroke onset and it is delivered during hospitalization, but no longer than 4 weeks post stroke. The primary outcome is hand dexterity assessed by the Box and Block Test; secondary outcomes are the Fugl-Meyer Assessment, hand grip strength, pinch strength, perceptual threshold of touch, degree of pain, and modified Rankin Scale score. Outcome measurements are conducted at baseline, post intervention and at 6-month follow-up.

**Discussion:**

Because of the wide inclusion criteria, we believe that the results can be generalized to the larger population of patients with a first-ever stroke who present with an upper limb paresis of varying severity. On the other hand, the sample size (*n* = 102) may preclude subgroup analyses in such a heterogeneous sample. The sham ESS treatment totals a mere 2% of the active ESS treatment delivered to the intervention group per ESS session, and we consider that this dose is too small to induce a treatment effect.

**Trial registration:**

ClinicalTrials.gov, NCT02250365. Registered on 18 September 2014.

**Electronic supplementary material:**

The online version of this article (doi:10.1186/s13063-017-1815-9) contains supplementary material, which is available to authorized users.

## Background

Stroke is ranked as the third largest cause of disease burden globally [1], causing substantial physical, psychological and financial demands on patients, families, and societies at large [2–4]. Upper limb paresis is one of the most frequent impairments following stroke and affects 48–77% of patients in the acute stroke phase [5–7]. Moreover, upper limb paresis has been identified as a major obstacle to regaining independence in activities of daily living (ADLs) [8]. In fact, only 12–34% of the patients achieve full functional recovery of the affected upper limb at 6 months post stroke [9, 10]. This represents a considerable challenge since near complete functional recovery is required to routinely involve the affected upper limb in performing ADLs [11].

Recovery of upper limb functioning is typically pronounced during the first month and subsequently levels off by 6 months post stroke [12–14]. Regaining hand dexterity (i.e., motor skills such as reaching, grasping, gripping, moving and releasing objects) is often achieved already within the first 4 weeks, implying that there may be a critical time window for recovery of upper limb functioning [9, 10] during which rehabilitation efforts may maximize functional recovery. However, there are few studies investigating the effect of motor rehabilitation methods in the initial weeks after stroke.

Electrical stimulation (ES) is one of the methods that have been used to facilitate recovery of upper limb functioning following stroke. ES can induce a muscle contraction, or it can be a somatosensory stimulation below the motor threshold [15]. The majority of studies using ES have been conducted in chronic stroke and, therefore, it remains unknown to what extent ES applied in the acute phase after stroke could affect the recovery of upper limb functioning. Also, these investigations have largely focused on ES that induces muscle contraction. In healthy persons, the application of low-intensity ES with no or small motor responses to peripheral hand nerves [16–20], forearm muscles [21] or the whole hand [22, 23] elicits an increase in the cortical excitability of the representations that control the stimulated body parts, which seems to outlast the stimulation period itself [18, 21, 23]. It has been hypothesized that increasing the amount of somatosensory input may enhance the motor recovery of patients following stroke [24]. Recent data on acute, subacute and mostly chronic stroke patients suggest that a single 2-h session of ESS to the peripheral hand nerves leads to transient improvement of pinch force, movement kinematics and upper limb motor skills required for ADL performance [25–31]. However, ESS was only used in conjunction with motor training in one of these studies [29]. Interestingly, there is some evidence that multiple sessions of ESS to the peripheral hand nerves, in conjunction with motor training, might improve motor skills of the paretic upper limb in subacute [32, 33] and chronic stroke patients [34], and, moreover, that these positive results seems to be long lasting [34]. However, the effect of ESS in conjunction with motor training has never been investigated in acute stroke patients. It is noteworthy that ESS is benign in nature, causes patients minimal discomfort and adverse effects (itch and blushing), is relatively inexpensive and can easily be incorporated into clinical practice [35]. Therefore, it would be valuable to establish the effect of multiple sessions of ESS in conjunction with motor training in the restoration of upper limb functioning in the acute stroke phase.

The purpose of the present trial is to investigate the effect of multiple sessions of ESS treatment accompanied by motor training on the recovery of the affected upper limb following stroke. The ESS treatment is initiated in the acute stroke phase and each ESS session is immediately followed by motor training of the paretic upper limb. Specifically, we wish to address the following:

1. Does ESS treatment: (a) reduce motor and sensory impairments, (b) improve hand dexterity and (c) reduce disability at the end of the intervention period (short-term effect)?
2. Are the changes that can be observed at the end of the intervention period still present or improved at 6 months post stroke (long-term effect)?

Our hypothesis is that ESS treatment initiated in the acute stroke phase will improve paretic upper limb functioning as measured by the Box and Block Test (BBT) (primary outcome measure) at 6 months post stroke.

## Methods/design

### Trial design

**Fig. 1:**
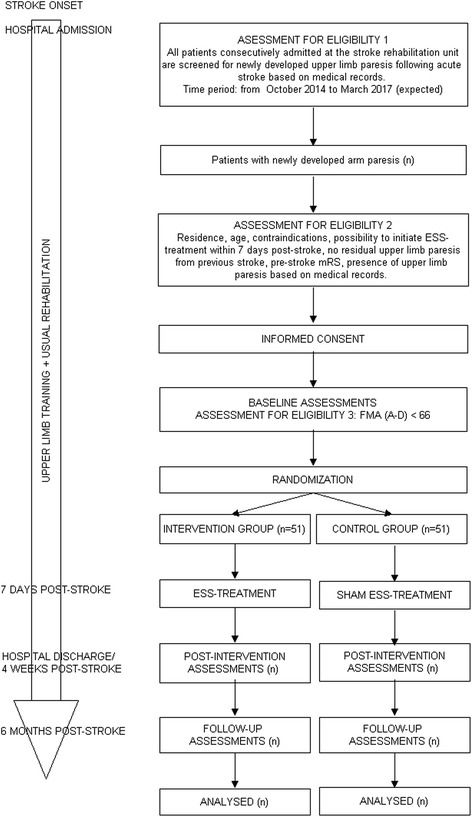
SPIRIT flow diagram of the trial

This study is conducted as a single-blinded randomized controlled trial with two arms and blinded endpoint adjudication. The intervention consists of ESS/sham ESS treatment immediately followed by training of the affected upper limb in addition to usual rehabilitation. The ESS/sham ESS treatment is initiated within the first 7 days post stroke. The first 4 weeks post stroke seem to be crucial for gaining maximal recovery of upper limb functioning [9, 10]. Since it is not possible for us to continue the intervention after hospital discharge for financial and logistical reasons, we decided to investigate the effect of our intervention during hospital stay (mean hospital stay when designing this study: 21 days), but no longer than 4 weeks post stroke. Outcome measures are assessed at three time points: (1) within the first 7 days post stroke prior to intervention onset (baseline), (2) at hospital discharge or 4 weeks post stroke (post-intervention) and (3) at 6 months post stroke (follow-up), which is the time point where the recovery of upper limb functioning is expected to level off [12–14]. Figure 1 shows the SPIRIT flow diagram of the trial.

### Trial setting and participants

Trial participants are recruited among patients admitted to the stroke unit (consisting of an acute unit and a rehabilitation unit) at Bispebjerg Hospital, which is one of the university hospitals in the Capital Region of Denmark. The hospital currently serves a well-defined urban catchment area with a population of approximately 400,000 citizens from the Municipality of Frederiksberg and the larger part of Municipality of Copenhagen for stroke rehabilitation [36]. Except for holiday periods and periods of recruitment of new trial personnel, all patients consecutively admitted to the stroke unit are assessed for eligibility by EG according to following inclusion criteria:

1. Admission at the rehabilitation stroke unit
2. Diagnosis of acute stroke confirmed by magnetic resonance imaging (MRI) or computed tomography (CT) scan
3. Upper limb paresis as indicated by a subscore <66 on the upper limb section of the Fugl-Meyer Assessment (FMA) (A–D) (see “Outcome measures” section)
4. Residence in the hospital’s catchment area
5. Aged 18 years or older

Patients are not included if any of the following exclusion criteria are present:

1. Contraindications to ESS (e.g., pacemaker in situ, skin impairment) [37]
2. Inability to initiate the ESS treatment within 7 days post stroke
3. Presence of cognitive dysfunctions or poor communication skills in Danish that limits the ability to provide informed consent
4. Severe prestroke disability as indicated by a modified Rankin Scale (mRS) score = 5 (see “Outcome measures” section)
5. Incomplete recovery of the affected upper limb after a previous stroke
6. Participation in other biomedical intervention trials within the last 3 months

The recruitment process takes place in several steps which are presented in the flow diagram in Fig. 1.

We are collecting data on the following variables to characterize the sample and to assess whether the randomization is successful: (a) age, (b) gender, (c) profession, (d) living condition (i.e., married or living with another person, alone), (e) risk factors for stroke: smoking, alcohol consumption, prestroke nutritional habits, prestroke physical activity level, weight, height, Body Mass Index, waist circumference and other risk factors for stroke (e.g., previous stroke, hypertension, hyperlipidemia, heart disease), (f) type of stroke (i.e., hemorrhage or infarction), (g) subtype of ischemic stroke [38], (h) severity of stroke at baseline [39], (i) stroke acute treatment, (j) affected upper limb, (k) dominant hand, (l) medications, (m) complications during hospitalization, (n) amount and content of upper limb training following ESS and (o) amount and content of usual rehabilitation. For further information, see Table 1.

**Table 1:** Time schedule of enrollment, intervention, assessments and responsible trial personnel

Trial procedure	Time points during the course of the trial					Responsible personnel	Blinded to group allocation?
	Stroke onset – 7 days post stroke	ESS treatment	Post- intervention	Hospital discharge	6-month follow-up		
Assess eligibility	X					EG	Not applicable
Present trial information for potential participants	X					EG/ESS personnel	Not applicable
Collect informed consent	X					EG	Not applicable
Group allocation	X					Administrative personnel/SPM	No/no
Assess outcome	X		X		X	EG	Yes
Collect sociodemographic data	X			X	X	EG	Yes
Collect medical data	X			X	X	MS/EG/ESS personnel	Yes/yes/no
Collect data on prestroke nutritional habits and physical activity	X					EG/ESS personnel	Yes/no
Collect data on upper limb training and usual rehabilitation		X				OTs or PTs at the stroke unit and in the community	Yes
Collect data on ESS treatment, including adverse events		X				ESS personnel	No

*EG* Emma Ghaziani, *ESS* electrical somatosensory stimulation, *MS* Mette Søndergaard, *OT* occupational therapist, *PT* physiotherapist, *SPM* S. Peter Magnusson

### Intervention

**Fig. 2:**
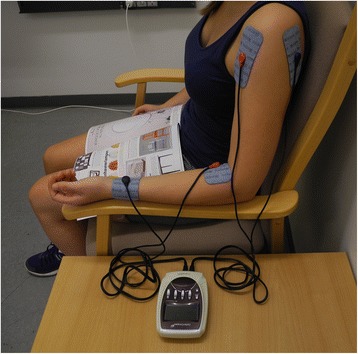
Placement of the electrodes

The ESS treatment is administered at the rehabilitation stroke unit. Trained health care personnel who are not involved in usual rehabilitation or outcome assessments initiate the ESS treatment within 7 days post stroke according to a standardized protocol. Both groups receive 1 h of daily ESS from Monday to Sunday throughout their hospital stay, but for no longer than 4 weeks post stroke. Cortical excitability in healthy persons increases after 2 h of suprasensory ESS of peripheral hand nerves at the wrist [18, 20], but it is sufficient with 30 min of suprasensory ESS of the whole hand to induce increased cortical excitability that lasts for 1 h after stimulation has ended [22, 23]. When electrical stimulation is applied for a longer time period, it seems that cortical excitability reaches a plateau after 45 min [19] and, therefore, we decided to provide the ESS treatment for 1 h. A Cefar Compex Theta 500 electrical device specially programmed to deliver ESS/sham ESS is used (DJO Global Switzerland Sàrl, Ch. du Dévent, Z.I. Larges Pièces A, 1024 Ecublens, Switzerland). Two sets of large electrodes (Performance electrodes, 10 × 5 cm, One Snap, DJO Nordic, AB., Murmansgatan 126, 212 25, Malmö, Sweden) are placed on the affected arm as follows (see Fig. 2):

1. One set of electrodes on the upper arm – one electrode on the front of the shoulder and the other on the back of the shoulder, both of them covering the lower part of the deltoid muscle
2. One set of electrodes on the forearm – one electrode just distal to the elbow crease, centered on the anterior aspect of the forearm; the other electrode just proximal to the palm of the hand on the anterior and radial aspect of the forearm

The stimulation level is determined individually for each patient in the beginning of each ESS session. Halfway into each ESS session, the stimulation level is adjusted, if necessary, in the intervention group, whereas the participants in the control group receive a short visit from the ESS personnel in order to ensure that the same amount of attention is given to both groups.

The intervention group receives suprasensory ESS delivered in a continuous mode (pulse width = 250 μs, frequency = 10 Hz). Suprasensory ESS is defined as the highest current amplitude that elicits paresthesia without any discomfort, pain or visible muscle twitches. Sham ESS is suprasensory ESS delivered in an intermittent mode (active stimulation intervals of 3 s are delivered in loops of 2.5 min, pulse width = 250 μs, frequency = 10 Hz). In this way the control group receives a total dose corresponding to 2% of the amount of active ESS delivered to the intervention group per ESS session, and we consider this dose to be too small to induce treatment effect. Apart from ESS/sham ESS during the hospital stay, no other electrical therapy is permitted to the affected upper limb during the 6-month trial period. Delivering other types of interventions (e.g., cognitive or motor training) is not allowed during the ESS sessions. For further details, see the ESS treatment protocol in Additional file 1.

The ESS/sham ESS treatment is followed by motor training of the affected upper limb that takes place within 30 min after cessation of the ESS. We expect that brain excitability will be increased during this time interval due to the ESS [18, 23]. To our knowledge, there is no commonly acknowledged protocol for motor training of upper limb paresis following stroke and, therefore, we decided that the motor training would be provided in accordance to the existing clinical practice at the department which is currently not standardized. Thus, the upper limb training consists of active, repetitive, task-oriented practice. If the trial participant presents a severe upper limb paresis with no or highly limited active movements, the therapist in charge for the particular training session decides which other intervention methods will be employed. A task-oriented exercise bank (see Additional file 2) is available for the physical (PTs) and occupational therapists (OTs) delivering the upper limb training. However, it is a requirement that trial participants receive a minimum of 15 min of arm training following each ESS session. Both groups are offered the same upper limb training.

Usual rehabilitation is defined as PT and OT training received by the stroke patients in the hospital as well as at rehabilitation centers and, if relevant, at nursing homes in the community during their 6-month trial participation.

### Outcome measures

The primary outcome measure is the Box and Block test (BBT) at 6 months post stroke. The BBT assesses the upper limb’s capacity to perform reaching, grasping, moving and releasing objects unilaterally [40]. These motor skills are essential components of performing ADLs and, therefore, of relevance when evaluating whether our intervention contributes to achieving the ultimate goal of rehabilitation: independence in daily life. Normative data are available for the healthy adult population [40], and the instrument has been validated for use in stroke patients [41].

The secondary outcomes measures are: (1) the upper extremity section of the Fugl-Meyer Assessment (FMA-UE) [42–44], (2) hand-grip strength [45], (3) palmar, lateral and thumb-to-index pinch strength [45], (4) perceptual threshold of touch [46, 47], (5) pain in the upper limb during performance of the BBT using Numerical Rating Scale-11 [48] and (6) the mRS score [49, 50]. The FMA-UE, hand-grip strength, pinch strength, perceptual threshold of touch, and degree of pain are ways to quantify the level of motor and sensory impairments in the upper limb. The mRS is a clinician-reported measure of global disability which is widely used as an endpoint in stroke trials.

All outcome measurements are carried out by EG. Baseline assessments are performed at the stroke unit. Post-intervention and 6-month follow-up assessments are performed at the stroke unit, the patient’s home, or the inpatient rehabilitation center or nursing home, depending on the residence of the participant at the scheduled time.

### Sample size estimation

Using a pretrial power analysis, we determined that a minimum sample size of 37 patients was required for correctly detecting a within-group improvement of 5.5 on the BBT, if such a difference truly exists, with a two-sided significance level of 5% and a power of 80% [51]. The number of 5.5 blocks had previously been reported as the smallest real difference between two measurements for the affected upper limb [41]. Since the minimally clinically important difference on BBT is, to our knowledge, not established yet, we made the assumption that the smallest real difference would be perceived by stroke patients as being of clinical relevance for their daily life. After adjusting for: (a) a case-fatality rate of 8% as reported at 1 year post stroke in a Danish nationwide population-based study [52] and (b) a 20% loss of participants at follow-up for other reasons, we estimated that 51 participants were needed in each group [53]. Hence, the total sample size is 102 participants.

### Randomization

Participants are allocated sequentially to either the intervention or the control group using a randomization list constructed by block randomization with variable block size. Randomization is furthermore stratified on: (a) sex and (b) the ability to perform active finger extension at baseline; active finger extension has shown to be a simple and reliable early predictor of recovery of upper limb functioning in stroke patients [9, 54]. The randomization sequence was generated with the random generator in SAS. The randomization list is kept by administrative personnel and concealed from the other project investigators, with the exception of SPM. Allocations are performed after baseline assessments by contacting the administrative personnel who forwards the group allocation to the personnel responsible for ESS/sham ESS treatment. SPM is occasionally involved in performance of group allocation in the absence of the administrative personnel.

### Blinding

Although complete blinding of the participants to the group allocation is impossible because of the nature of the ESS treatment (i.e., participants can feel the stimulation and are aware of whether it is delivered in continuous or intermittent sham mode), participants are kept unaware of how it is supposed to feel. The personnel who apply ESS are not blinded to the group allocation, and they also collect data from medical records that will be used to characterize the sample. The therapists providing usual rehabilitation as well as other personnel involved in usual patient care are blinded to the group allocation. Investigators who perform outcome assessments and data analysis are unaware of the group allocation, with the exception of the principal investigator (SPM) who is involved in group allocation in the absence of the administrative personnel, who are normally responsible for the group allocation and not involved in any other trial procedures. For further details see Table 1.

### Statistical analysis

Background characteristics will be compared between the intervention groups with *t* tests (continuous variables) or chi-squared tests (categorical variables). The development of both primary and secondary outcome variables will be analyzed in longitudinal models over the stroke recovery trajectory (baseline, post-intervention and 6-month follow-up), and the difference of the outcomes between the two intervention groups at each of the study time points will be analyzed in multivariable linear regression models. Analyses will be adjusted for the stratification variables (sex and the ability to perform finger extension). Possible differential attrition is adjusted for by weighting the outcomes that are available at each of the study time points with the inverse of the probability of being present; these probabilities are estimated in logistic regression models including all background characteristics and outcomes at previous study time points. To account for this weighting and for repeated observations on the same individual generalized estimating equations (GEE) methods are used to adjust the variance of the parameter estimates. Analyses are performed with SAS version 9.4. The statistical significance level is 5%.

Additional file 3 shows the SPIRIT Checklist [55, 56] for this study protocol.

## Discussion

To our knowledge, this is the first trial investigating the effect of multiple sessions of ESS treatment in the acute stroke phase on the recovery of upper limb paresis. Several studies have shown that the process of upper limb recovery, and especially that of hand dexterity, is most pronounced during the first 4 weeks post stroke after which the recovery gradually levels off before reaching a plateau around 6 months post stroke. Therefore, initiation of rehabilitation in the early weeks post stroke may be essential for achieving successful upper limb recovery at the end of the rehabilitation process. Our trial intends to evaluate the effect of ESS – a rehabilitation method with the potential of applicability in clinical practice. ESS is easy to administer, inexpensive, free of patient discomfort and probably highly acceptable to frail patients in the early days post stroke.

Contrary to previous studies that primarily focused on the stimulation of the peripheral hand nerves at wrist level, we use two sets of large electrodes (see “Intervention” section) to stimulate somatosensory receptors in the shoulder, elbow and wrist regions. We believe that the larger area covered may be beneficial, but because we do not have a third trial arm receiving ESS at wrist level only, we will not be able to identify the effect of increasing the stimulation areas per se.

The eligibility criteria for participation in this trial are very broad. Basically, we include all adult stroke patients living in the hospital’s catchment area, except those with remaining upper limb paresis from a previous stroke, contraindications to ESS, severe prestroke disability or inability to provide informed consent. Based on this nonrestrictive participant selection, the results can be generalized to the larger population of patients with a first-ever stroke or successful recovery after a previous stroke and who present an upper limb paresis of varying degrees of severity. However, since our trial is not powered to perform subgroup analyses, we may encounter challenges in detecting the effect of ESS in such a heterogeneous sample. The possibility of overlooking a treatment effect on a specific subgroup of stroke patients (type II error) is a limitation of the study.

Designing the control ESS treatment was challenging since the intervention can be perceived by the trial participants who may also interact with each other during hospitalization. Since a completely inactive ESS treatment gave rise to concerns about high dropout rates in the control group, we designed a sham ESS treatment with an extremely low treatment dose. It is unlikely that the total amount of sham ESS treatment (1.2 min) is sufficient to induce any training effects. However, we are unable to verify this since we do not have a trial arm receiving completely inactive or no ESS. As a consequence, our trial results can be biased towards an underestimation of the effect of ESS treatment. It is noteworthy that both the control and the intervention group receive the same amount of attention from the personnel during the ESS sessions. Although some participants might figure out their group allocation, we hope that they would be motivated to continue in the trial due to fact that they are offered upper limb training in addition to the usual rehabilitation.

## Trial status

The recruitment of participants was initiated on 13 October 2014 and will continue until complete sample size is achieved which is expected in March 2017. At the submission time of this protocol article, patients are still being recruited for the trial.

## Additional files

Additional file 1: ESS treatment protocol version 2016_09_05. (DOCX 26657 kb)
Additional file 2: Exercise bank for active, repetitive, task-oriented upper limb training following stroke version 2017_01_22. (PDF 20460 kb)
Additional file 3: The SPIRIT Checklist for the study protocol. (DOC 121 kb)

## Abbreviations

ADLs: Activities of daily living
ES: Electrical stimulation
ESS: Electrical somatosensory stimulation
mRS: Modified Rankin Scale
OT: Occupational therapy/occupational therapist
PT: Physiotherapy/physiotherapist
