# Supplementary material for: Electrical somatosensory stimulation followed by motor training of the paretic upper limb in acute stroke: study protocol for a randomized controlled trial
Source: Trials. 2017 Feb 23;18:84. doi: 10.1186/s13063-017-1815-9 (PMC5324330; doi:10.1186/s13063-017-1815-9)
Supplement: Additional file 1: — ESS treatment protocol version 2016_09_05. (DOCX 26657 kb) [file 13063_2017_1815_MOESM1_ESM.docx]

**ELECTRICAL SOMATOSENSORY STIMULATION (ESS)**TREATMENT PROTOCOL

1. The subjects are given 60 minutes of either intermittent or continuous electrical somatosensory stimulation (ESS).
2. Prepare the subject by placing the four electrodes (1) on the paretic arm and connecting them to the ESS-device (2) using one cable for the shoulder electrodes and one cable for the electrodes on the forearm:


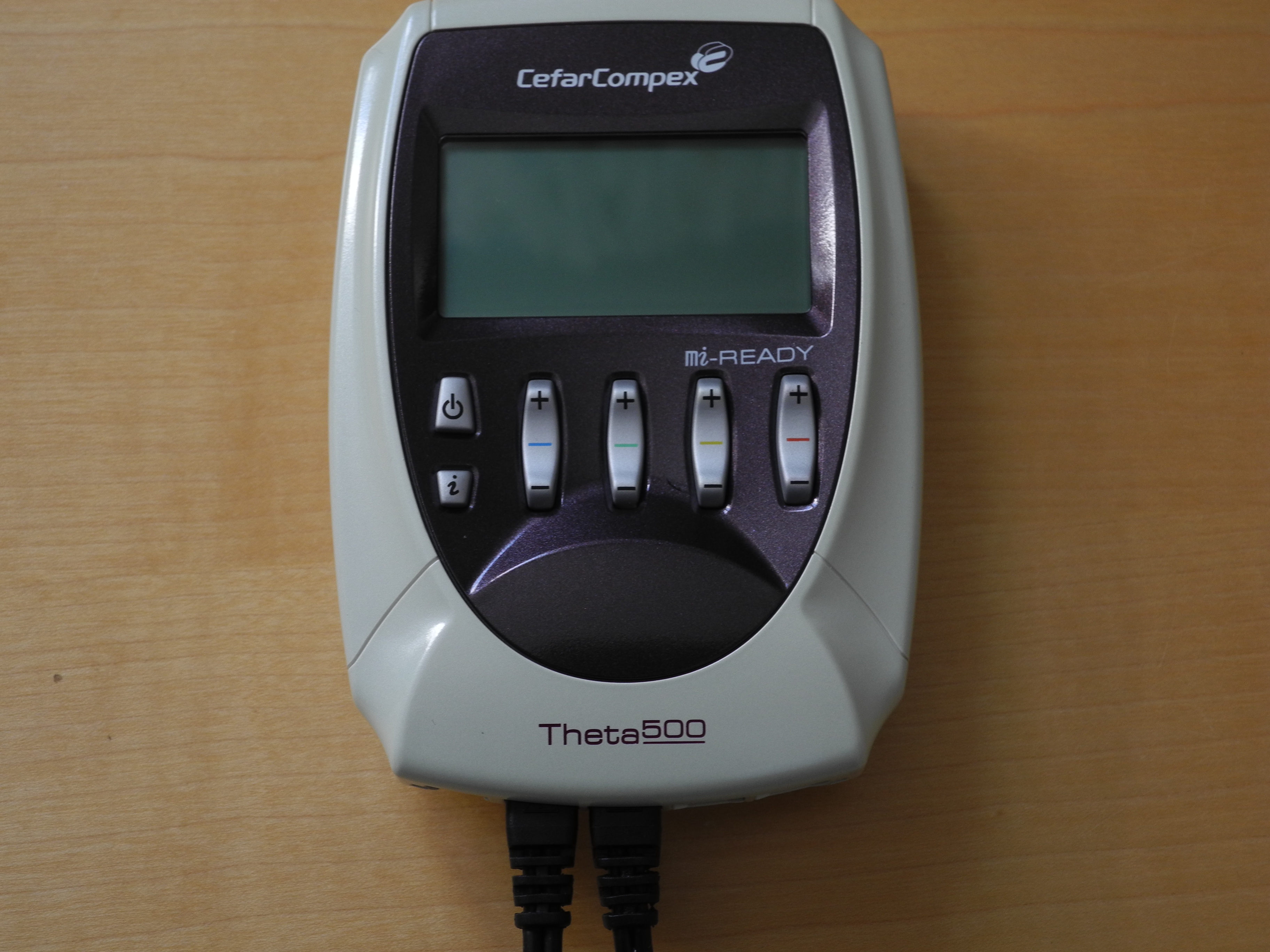

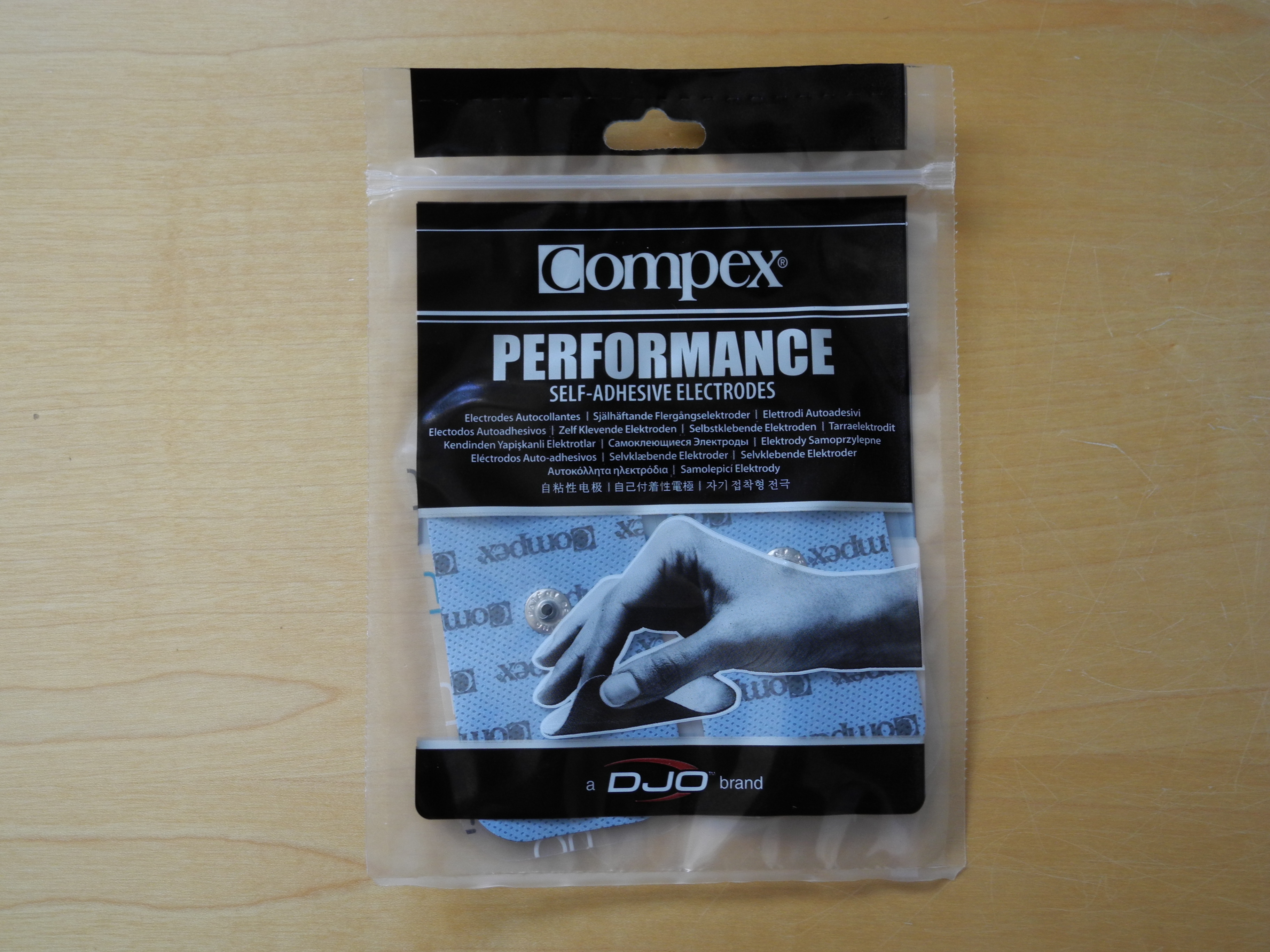


**1 2**


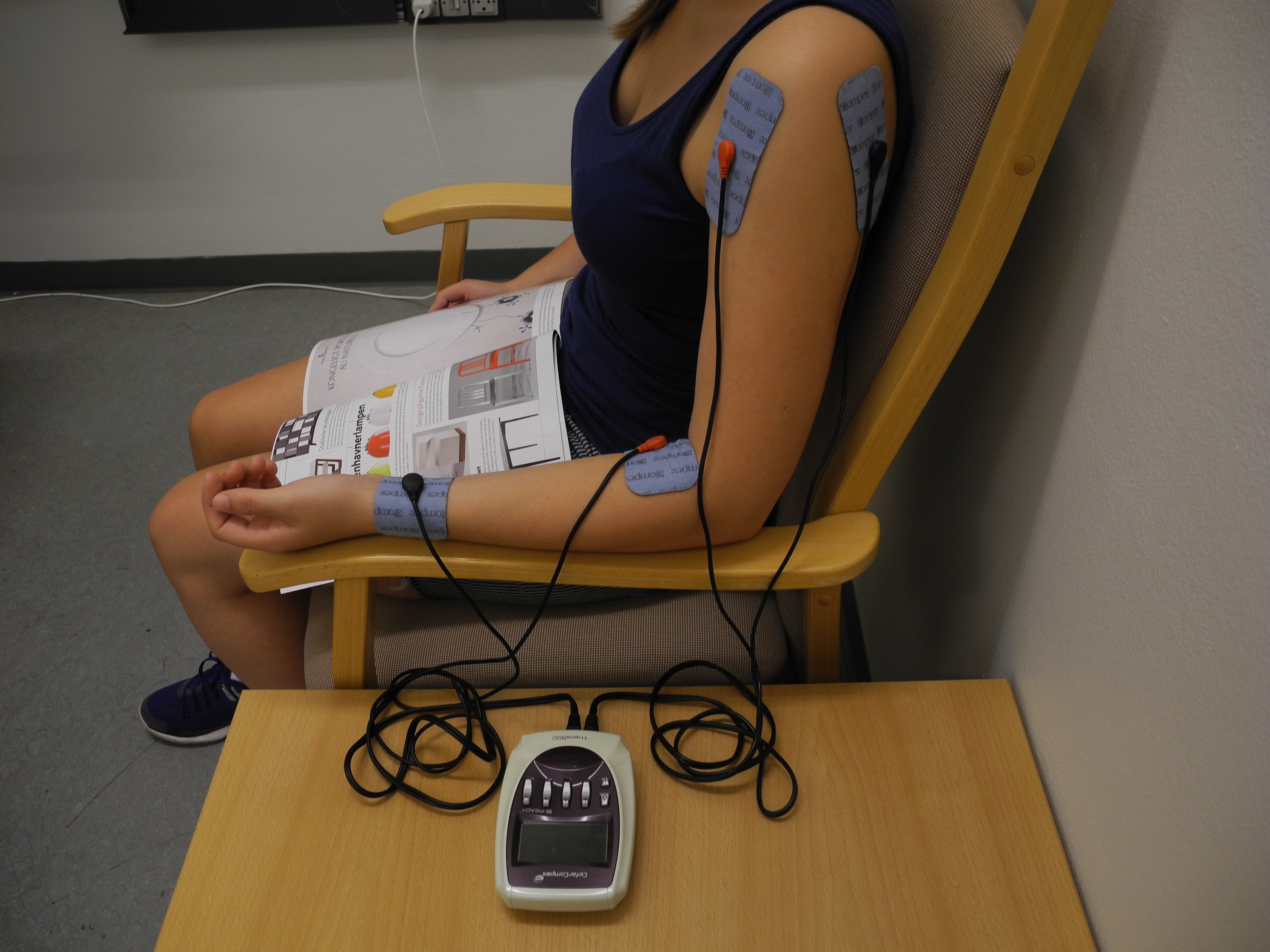


- 1. One of the shoulder electrodes is placed on the front of the shoulder and the other on the back of the shoulder, both of them covering the lower part of the deltoid muscle. The distance between them should at least correspond to the width of one electrode. The red part of the first cable is connected to the electrode on the front, and the black part of the cable to the electrode on the back of the shoulder:


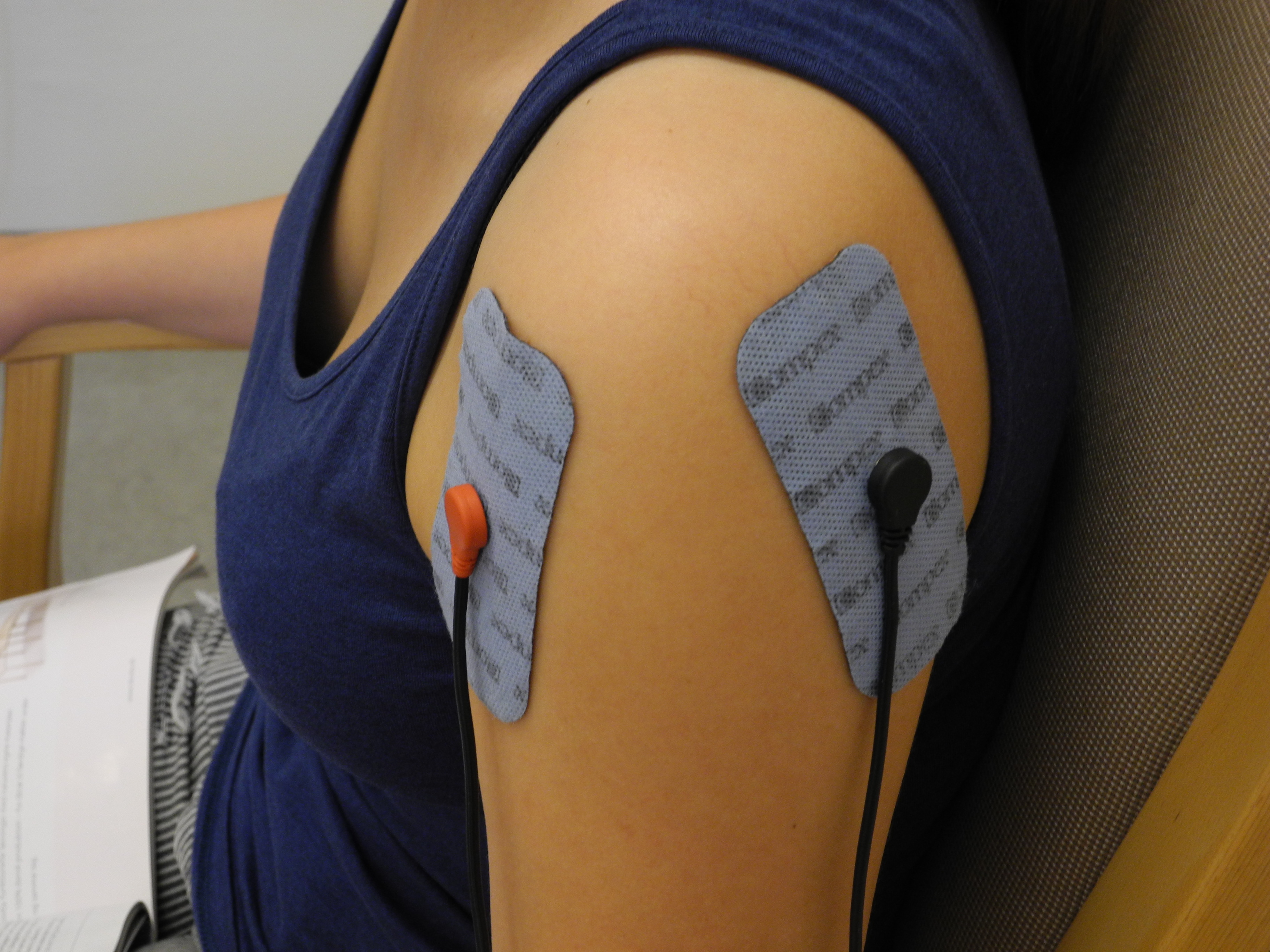


- 1. The elbow electrode is placed just distal to the elbow crease, centred on the anterior part of the forearm and is connected to the red part of the second wire:


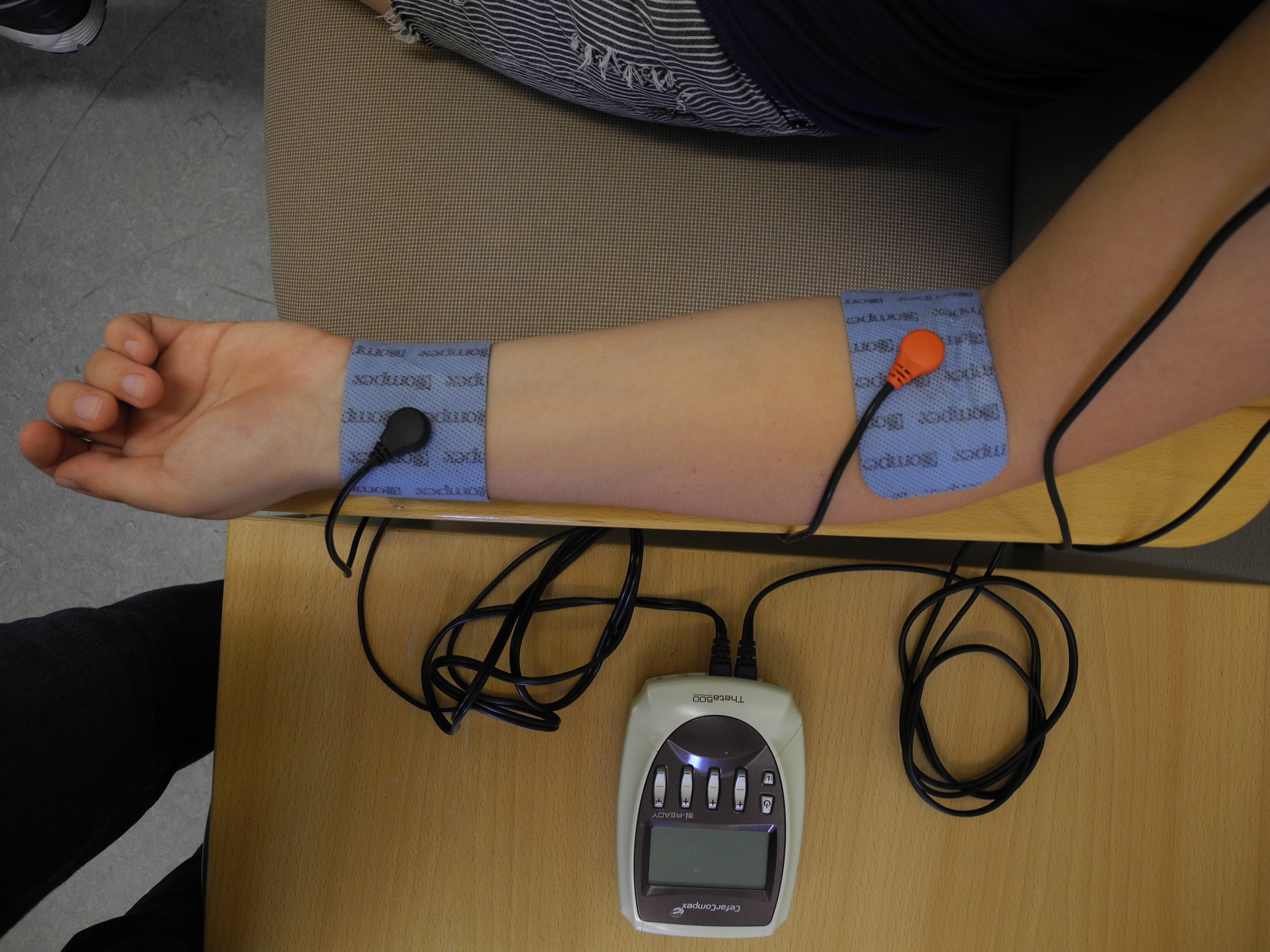


- 1. The wrist electrode is placed just proximal to the palm of the hand on the anterior and radial part of the forearm, covering part of the tabatière, and is connected to the black part of the second wire:


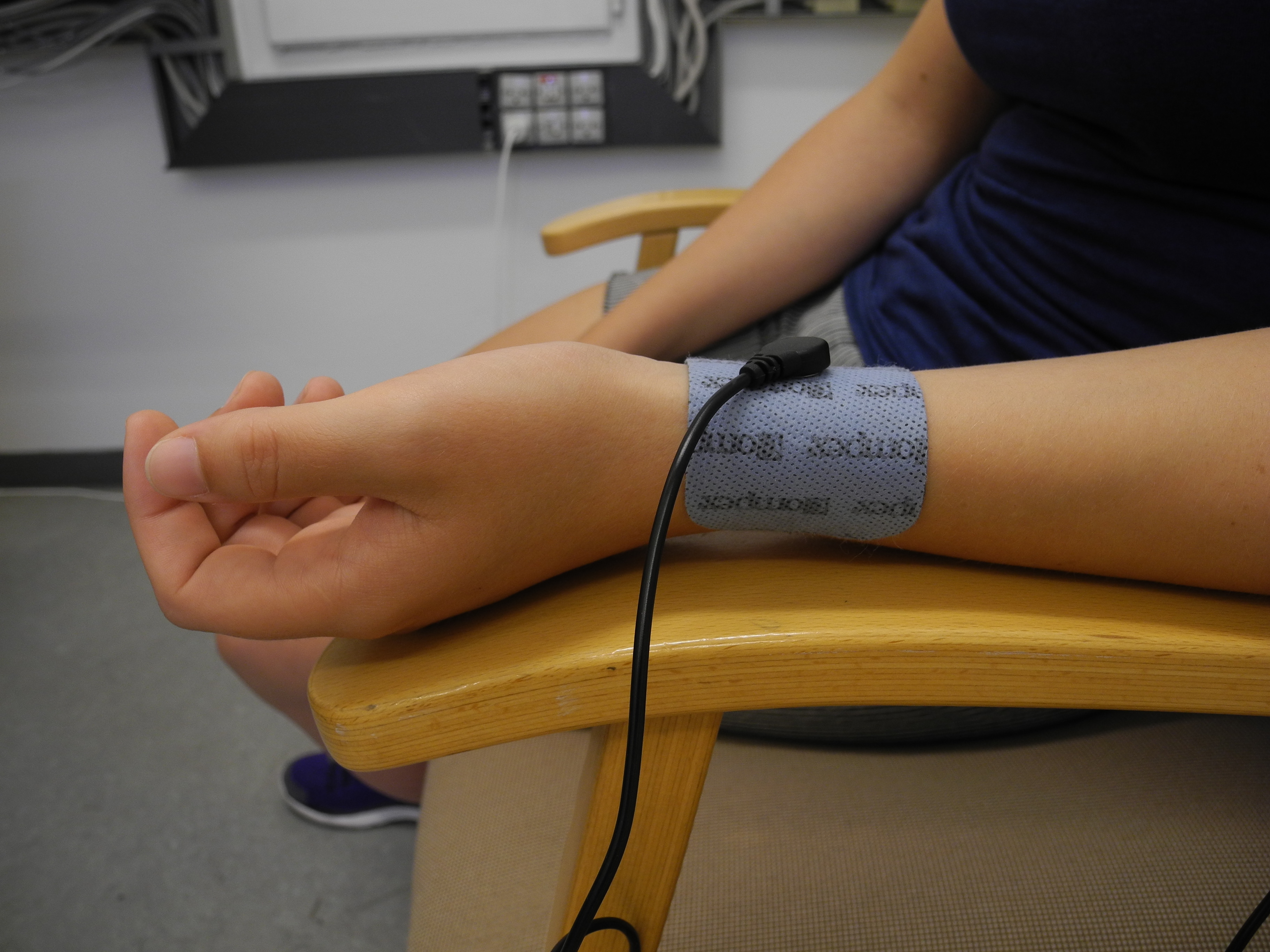


1. Ideally the subject should be supine with their arms in a relaxed position alongside their upper body. The forearm should be supinated with the dorsum of the hand resting on the bed in order to get a clear view of muscle twitches during the test. If it is not possible to position the patient like so, it is important to make sure the arm is as relaxed as possible. With some patients it will be necessary to support the arm in order to keep it from being fully pronated, which should by all means be avoided, as it will veil minor muscle twitches in the forearm and fingers.
2. Inform the patient about the treatment. Tell them that:
   1. They might feel the current as a tingling sensation in their arm and fingers.
   2. They are allowed to do normal everyday activities during the treatment but they should avoid all kinds of training of their paretic arm.
   3. They should tell you immediately if they feel any discomfort or pain at any given time.
3. Switch on the device and increase the strength of current in the forearm electrodes 1 mA at a time until small muscle twitches are seen in the forearm and/or fingers, or the patient signals discomfort or pain. Then decrease the strength of current until the discomfort or twitches disappear. Expectedly only a 1 mA decrease will be necessary.
4. Ask them if they feel the current in their 1^st^, 3^rd^ and 5^th^ fingers. If they do, continue by switching on the current in the shoulder electrodes in the same manner as with the forearm electrodes.
   1. If sensation is absent in one of the fingers, move the wrist electrode towards that finger (for the 1^st^ and 5^th^ fingers). Restart the device and find the suprasensory threshold again. If sensation is still absent in one of the fingers, note this in the document “T1: Registrering af ESS-behandling administreret af ESS-behandler” and carry on by switching on the current in the shoulder electrodes in the same manner as with the forearm electrodes.
5. After 30 minutes, return to the subject and ask if they are comfortable or are experiencing anything unforeseen. Check the cables, the electrodes and the device to make sure everything is still working.
   1. For subjects receiving continuous ESS, ask them to take the same position as at the beginning of the session. Try increasing the strength of current in both the forearm and the upper arm electrodes (one pair at a time). If pain or muscle twitches are induced, return to the initial strength of current. If it is possible to increase the strength of current without inducing twitches or discomfort, a new suprasensory threshold has been established and the treatment is continued at this level.
   2. For subjects receiving intermittent ESS, there will be no adjustment of the current after 30 minutes, but they will still be asked if they are comfortable or are experiencing anything unforeseen, and it is especially important to check the equipment with these subjects as they will have more difficulty in noticing an error.
6. After 60 minutes the treatment is over and the ESS-device switches off automatically. Before removing the electrodes, mark their position with a surgical skin marker to ensure similar positioning of the electrodes for the subsequent treatments.
7. Note the following in the document “T1: Registrering af ESS-behandling administreret af ESS-behandler”:
   1. The patient’s location
   2. If they have received 1 hour of ESS treatment
   3. The time of cessation of stimulation
   4. Side effects and/or notes if relevant
   5. Your name
