# Supplementary material for: Electrical somatosensory stimulation followed by motor training of the paretic upper limb in acute stroke: study protocol for a randomized controlled trial
Source: Trials. 2017 Feb 23;18:84. doi: 10.1186/s13063-017-1815-9 (PMC5324330; doi:10.1186/s13063-017-1815-9)
Supplement: Additional file 2: — Exercise bank for active, repetitive, task-oriented upper limb training following stroke version 2017_01_22. (PDF 20460 kb) [file 13063_2017_1815_MOESM2_ESM.pdf]

Bispebjerg and Frederiksberg Hospital
Department of Physical and Occupational Therapy

Research study: Electrical somatosensory stimulation followed by motor training of the paretic upper limb in acute stroke: a randomized controlled trial

Exercise bank for active, repetitive task-oriented upper limb training following stroke (version 2017_01_22)

This exercise bank is intended as inspiration for the PTs and OTs providing upper limb training to the trial participants in the above-mentioned research study.

The exercise bank is not exhaustive. Other similar exercises may be employed.

Task-oriented training is defined as performance of:

a) Isolated movements (e.g. flexion/extension of the wrist)
b) Exercises (e.g. moving objects from one location to another)
c) Parts of an ADL-task (e.g. using a spoon to fill oatmeal in a bowl)
d) A whole ADL-task (e.g. brewing coffee, getting dressed, eating)

Task-oriented training has a clear functional goal (e.g. pushing an object, moving objects from one location to another, getting dressed).

Each exercise should be individually adjusted to each trial participant's functioning level.

A high number of repetitions of the movement component or variations of the movement component to be trained should be performed.

Real-life object manipulation is preferred.

The inflatable plastic bandage (Urias bandage) is used to restrict degrees of freedom in the paretic upper limb.

In the table on the next page, the exercises are categorized according to the severity of upper limb paresis and the movement component that the exercise addresses.

REGION H

By: Rikke Mia Kastrup Mortensen, OT, the Stroke Department N10A/N11, Bispebjerg and Frederiksberg Hospital & Emma Ghaziani, PhD-stud, OT, Department of Physical and Occupational Therapy, Bispebjerg and Frederiksberg Hospital.
Translated into English by: Martin Bjørn Stausholm, MSc. in Physiotherapy, Department of Physical and Occupational Therapy, Bispebjerg and Frederiksberg Hospital.

	Severity of upper limb paresis	
**Movement components to be trained**	**Exercises for severe paresis (i.e. no active finger extension)**	**Exercises for mild to moderate paresis (i.e. active finger extension)**
Shoulder protraction/retraction	1, 8	14, 15, 21, 22, 40
Shoulder flexion/extension	2, 4, 5, 6	6, 11, 14, 15, 21, 22, 26, 27, 28, 35, 38, 39, 40
Shoulder abduction/adduction	7, 9	7, 11, 16, 20, 21, 22, 28, 38
Shoulder external rotation	10	10, 15, 16, 22, 28, 38
Elbow flexion/extension	4, 5	14, 15, 18, 20, 21, 22, 26, 27, 28, 40
Pronation/supination		17, 18, 19, 20
Wrist flexion/extension	3	14, 15
Radial/ulnar deviation of the wrist		23, 24
Grip: Finger flexion/extension		14, 15, 22, 24, 26, 27, 29, 30, 33, 37, 38, 39
Thumb abduction/adduction		12, 22, 26, 27, 33
Thumb flexion/extension		13, 36
Manipulating objects		23, 24, 28, 31, 32
Bilateral tasks		23, 28, 31, 32, 34, 35, 36, 37, 39
Other		25

References:

- Timmermans A, Spooren A, Kingma H, Seelen H. Influence of task-oriented training content on skilled arm-hand performance in stroke: a systematic review. Neurorehabilitation and Neural Repair. 2010;24(9):858-870.
- https://www.physiotherapyexercises.com

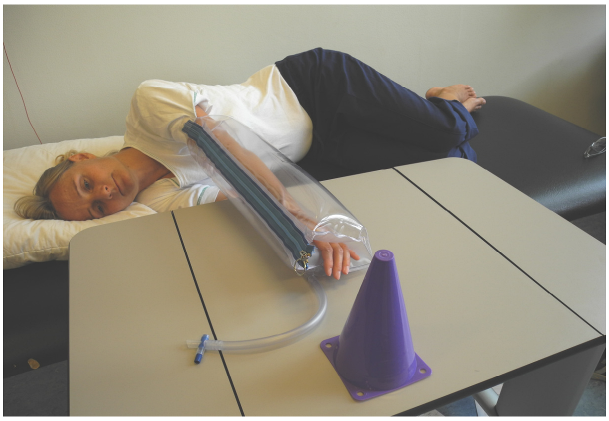

Exercise 1

- The patient pushes the cone by protracting the shoulder.

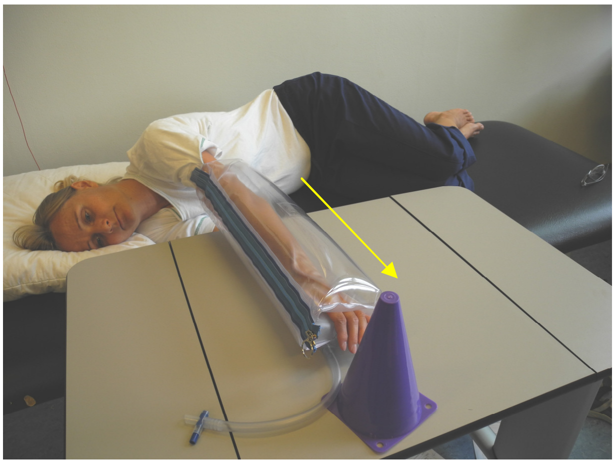

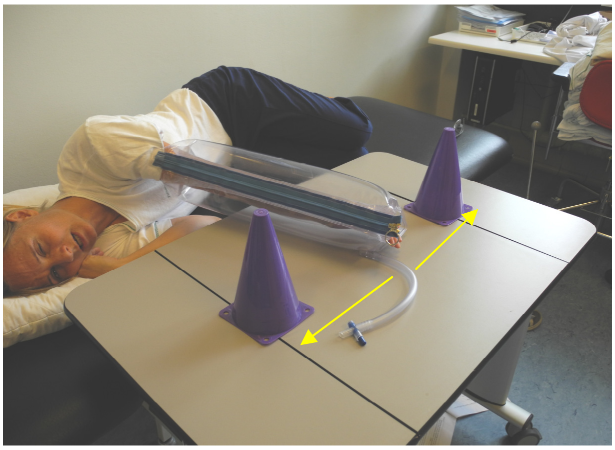

Exercise 2

- The patient pushes the cone by flexing/ extending the shoulder.

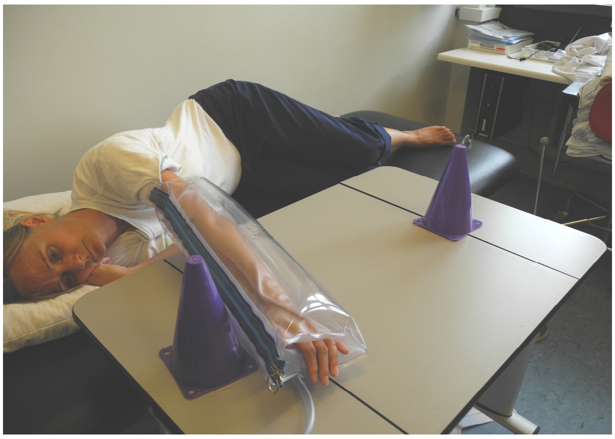

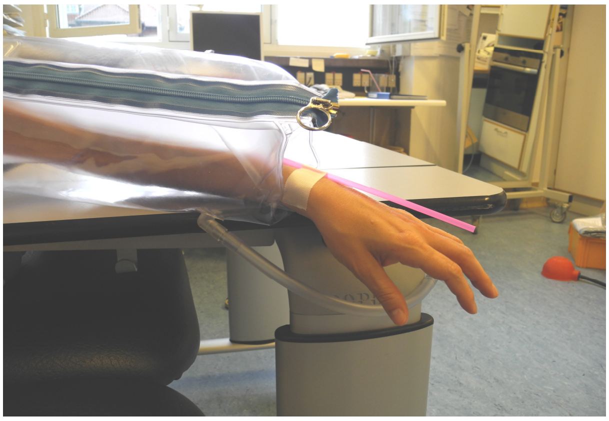

Exercise 3

- The patient's hand is placed in extended position and the patient holds the isometric contraction (i.e. the patient keeps touching the straw) for as long time as possible.
- Exercise progression: The patient aims for touching the straw by actively extending the wrist.

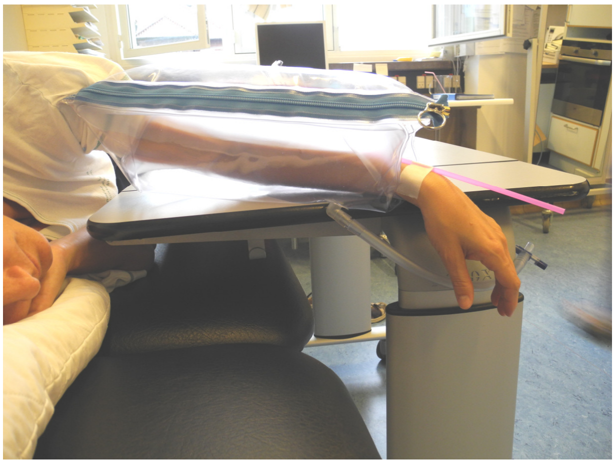

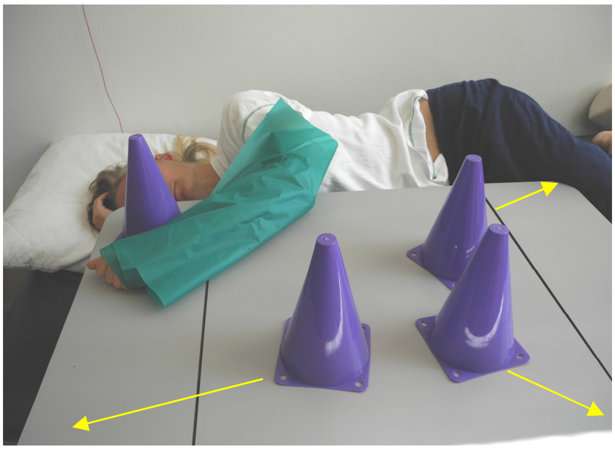

Exercise 4

- The patient extends/flexes the shoulder and the elbow to push the cones.
- The paretic arm can be wrapped in a material that diminishes friction.

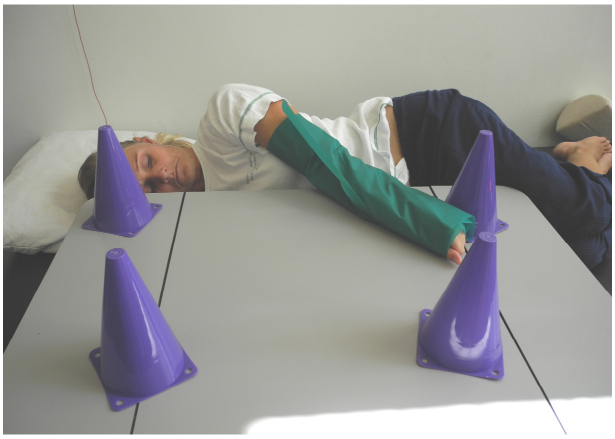

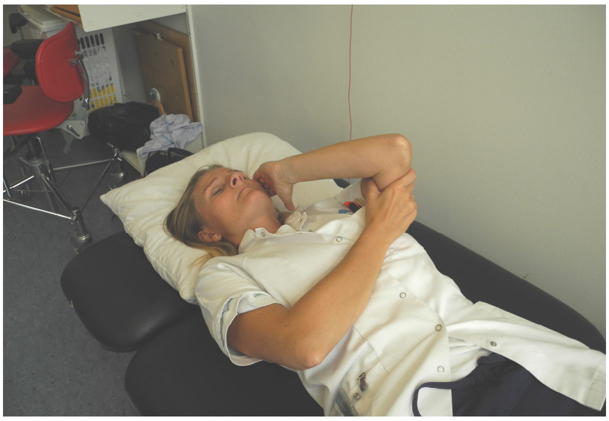

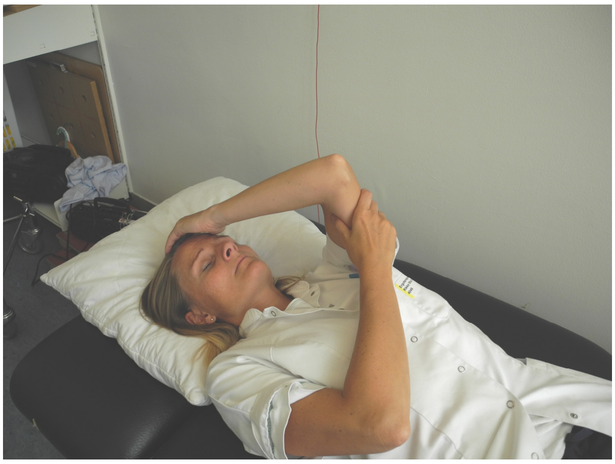

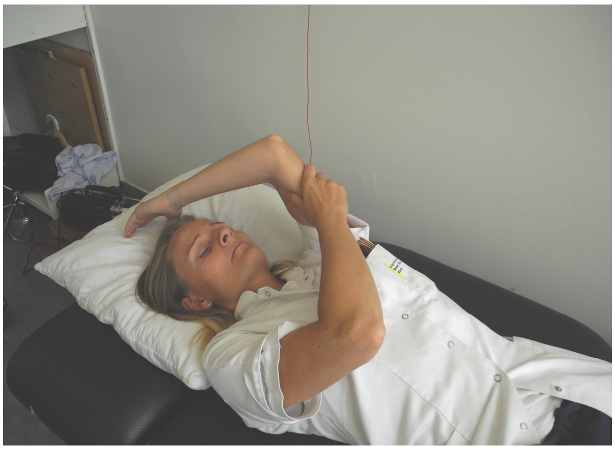

Exercise 5

- The patient touches the chin, hair or pillow with the paretic hand.

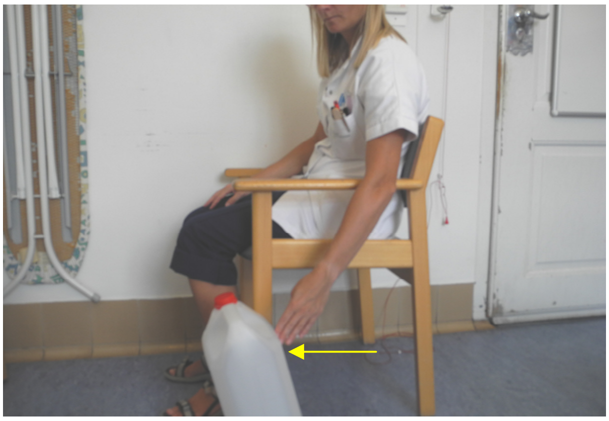

Exercise 6

- The patient overturns an object by flexing/extending the shoulder.
- The patient keeps the elbow extended. Urias bandage can be used to eliminate degrees of freedom in the elbow and wrist.

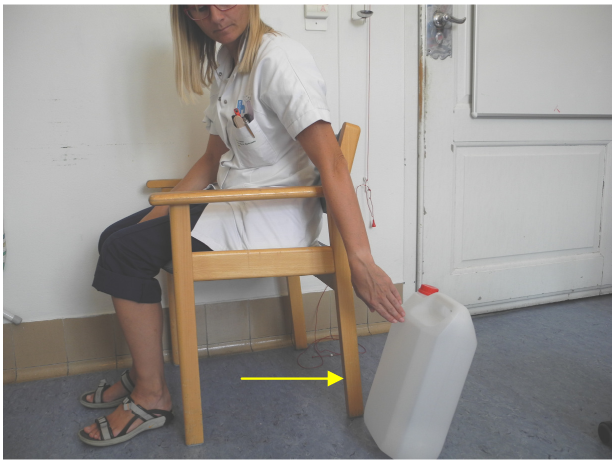

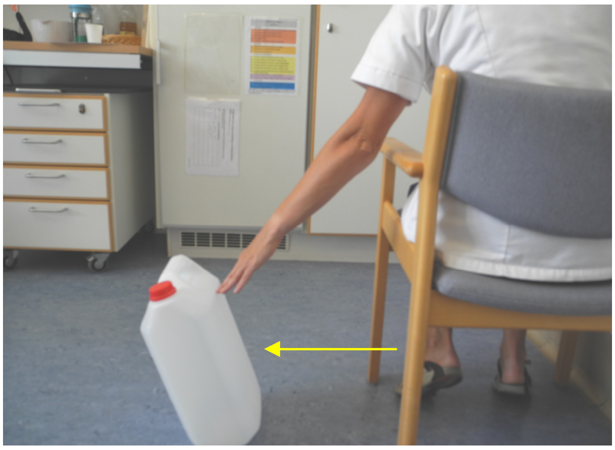

Exercise 7

- The patient overturns an object by abducting the shoulder.
- The patient keeps the elbow extended. Urias bandage can be used to eliminate degrees of freedom in the elbow and wrist.

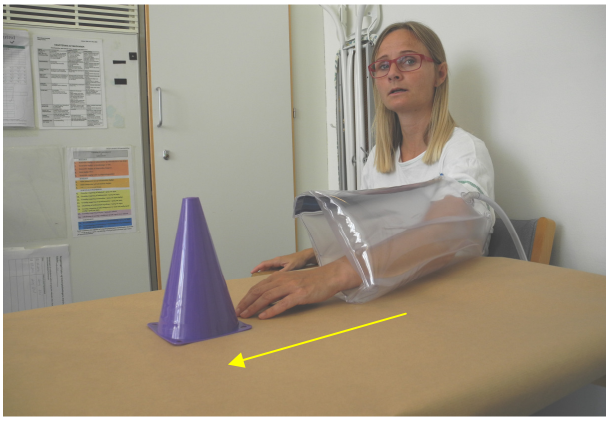

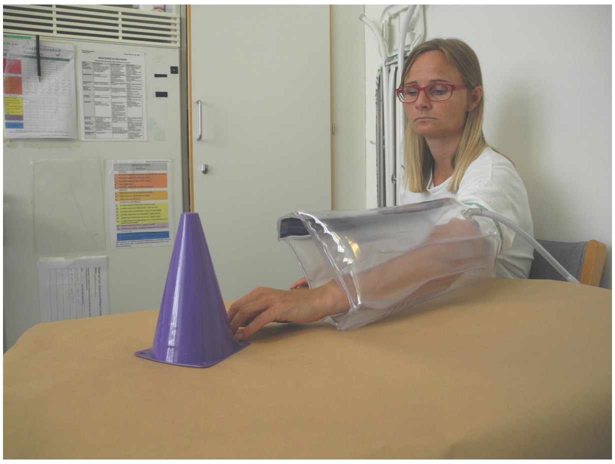

Exercise 8

- The patient pushes the cone by protracting the shoulder.
- Instead of using a cone, the therapist can draw a line on the table. The patient follows the line when protracting the shoulder.
- Urias bandage can be used to eleminate degrees of freedom in the elbow and wrist. The paretic arm can be wrapped in a material that diminishes friction.

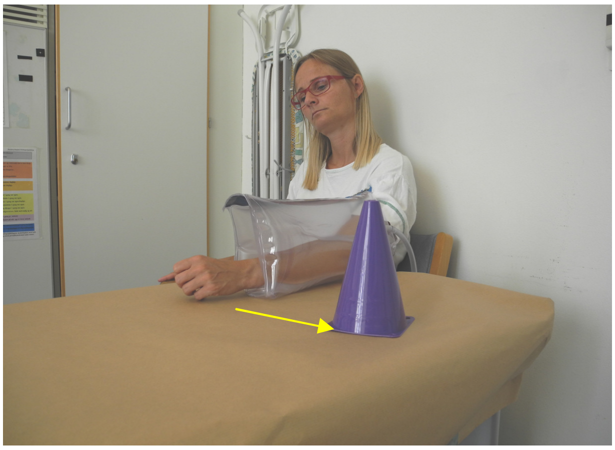

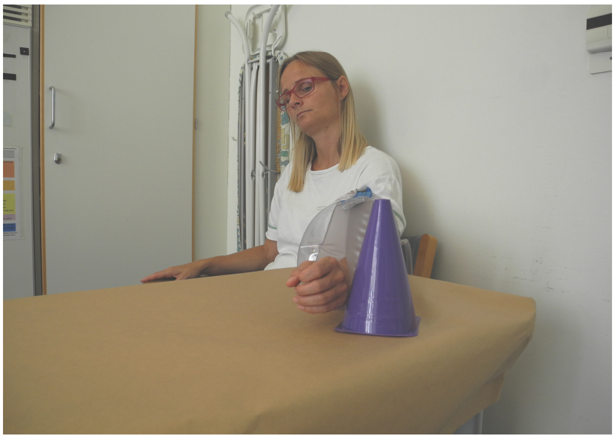

Exercise 9

- The patient pushes the cone by abducting the shoulder.
- Instead of using a cone, the therapist can draw a line on the table. The patient follows the line when abducting the shoulder.
- Urias bandage can be used to eliminate degrees of freedom in the elbow and wrist. The paretic arm can be wrapped in a material that diminishes friction.

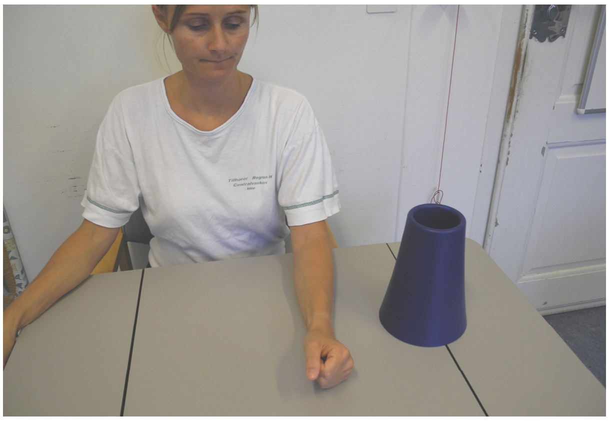

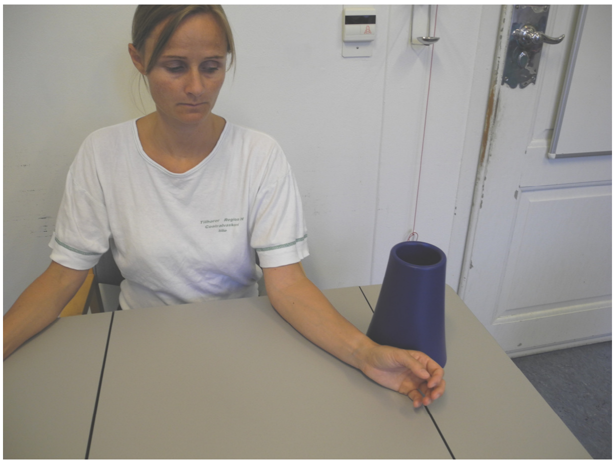

Exercise 10

- The patient pushes the cone by external rotating the shoulder.
- Instead of using a cone, the therapist can draw a line on the table. The patient follows the line when rotating the shoulder.
- The paretic arm can be wrapped in a material that diminishes friction.
- The patient may hold e.g. a dishtowel between the upper body and the upper arm to isolate the external rotation movement.

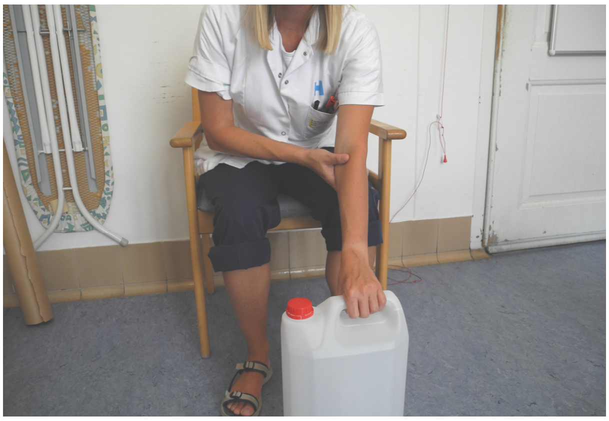

Exercise 11

- Flexion/abduction in the shoulder while holding a weight (e.g. a water tank).
- Exercise progression: Increase the weight (e.g. by filling more water in the tank).

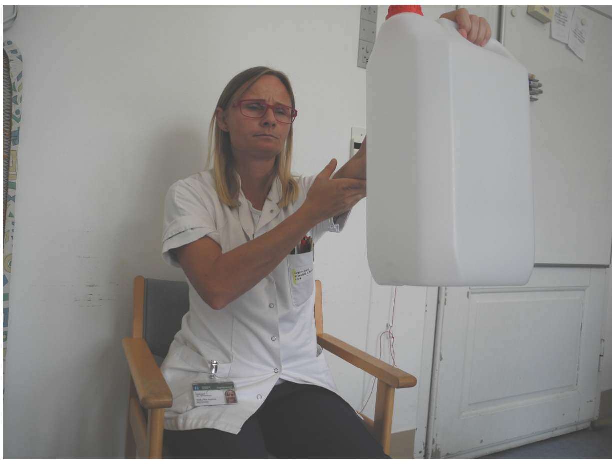

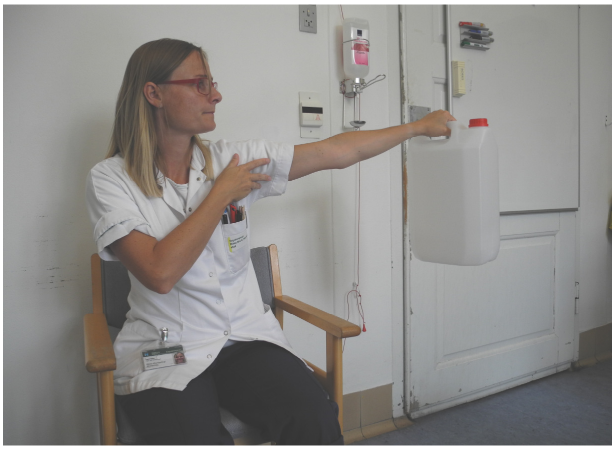

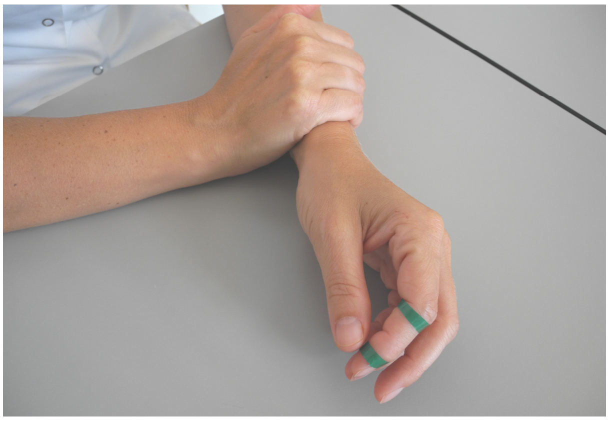

Exercise 12

- The patient adducts/abducts the thumb by moving the thumb between the two lines.

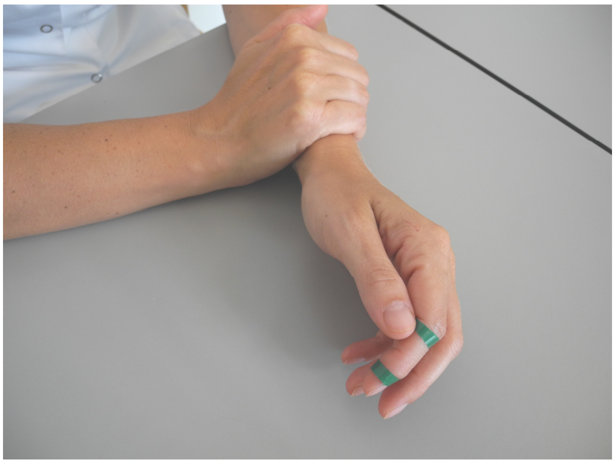

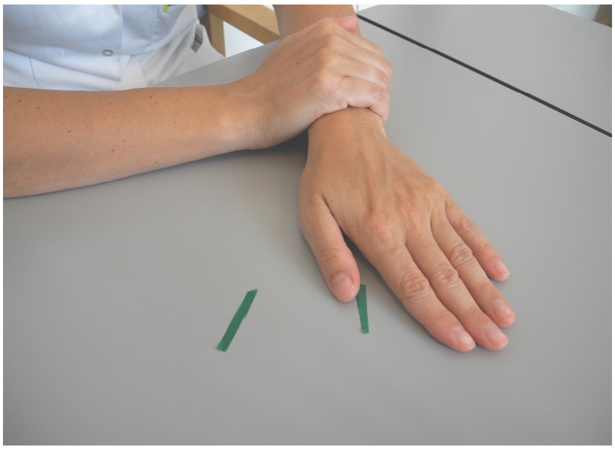

Exercise 13

- The patient flexes/extends the thumb by moving the thumb between the two lines.

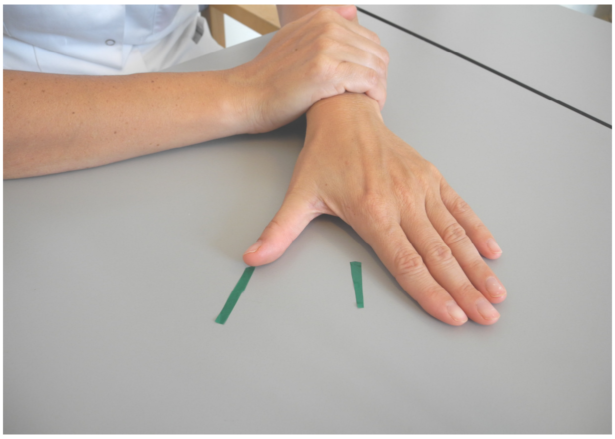

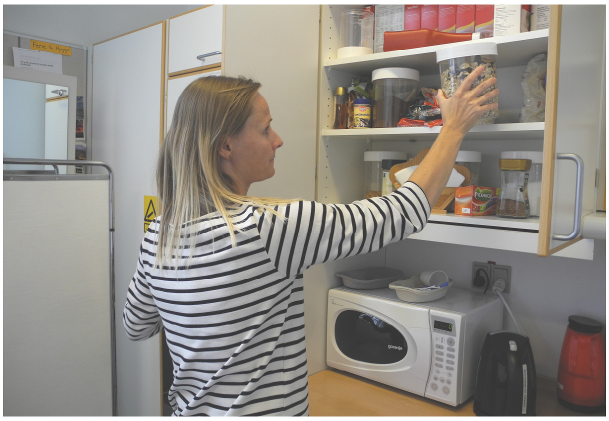

Exercise 14

- The patient takes down objects of different weights and shapes from a cupboard and places the objects on a surface in front of herself.
- The cupboard should be at eye level or higher.

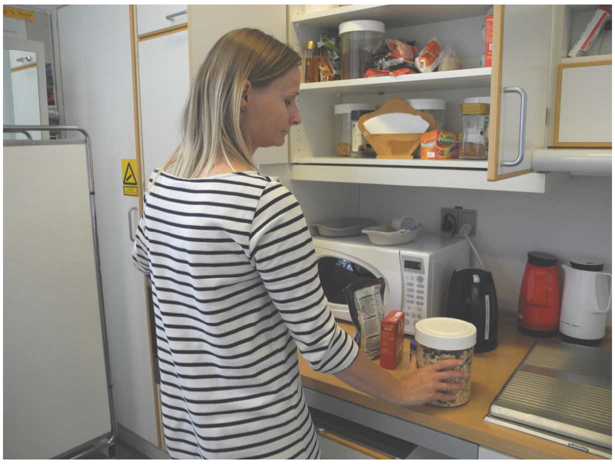

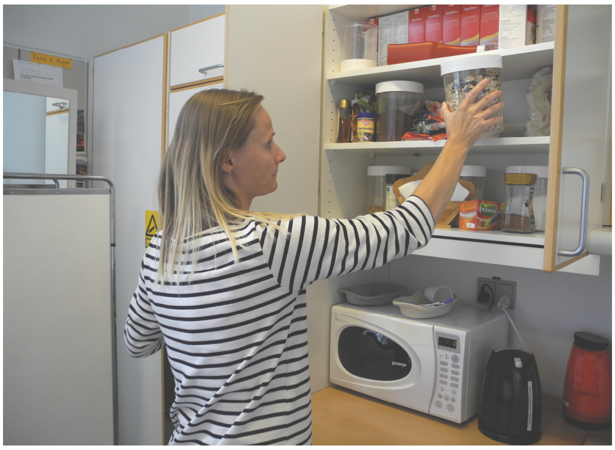

Exercise 15

- The patient takes down different objects of different weights and shapes from a cup-board and places them on a surface behind herself by rotating the upper body.
- The cupboard should be at eye level or higher.

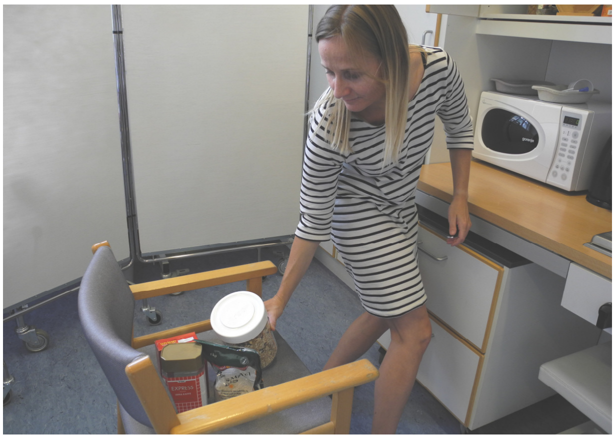

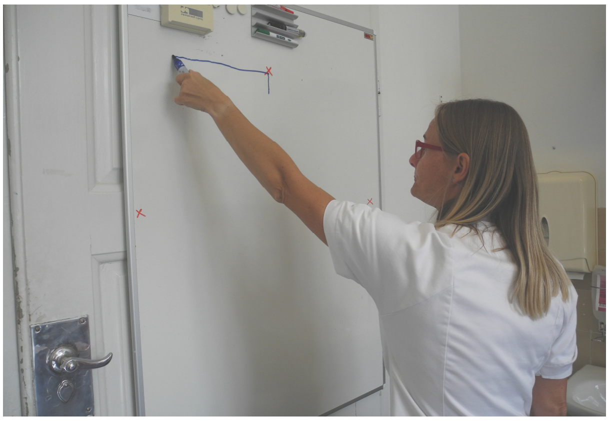

Exercise 16

- The patient trains extension, abduction and external rotation of the shoulder by drawing on a whiteboard.

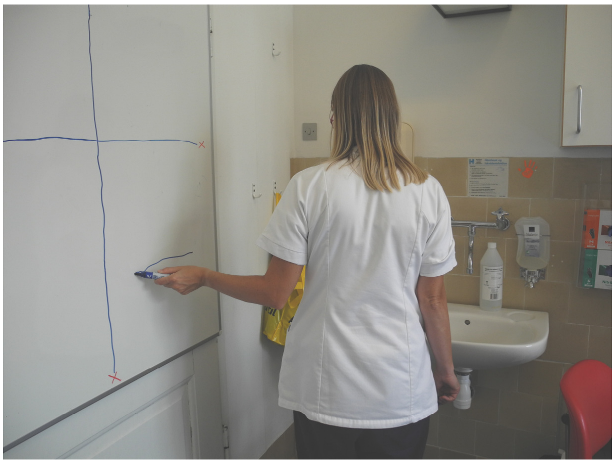

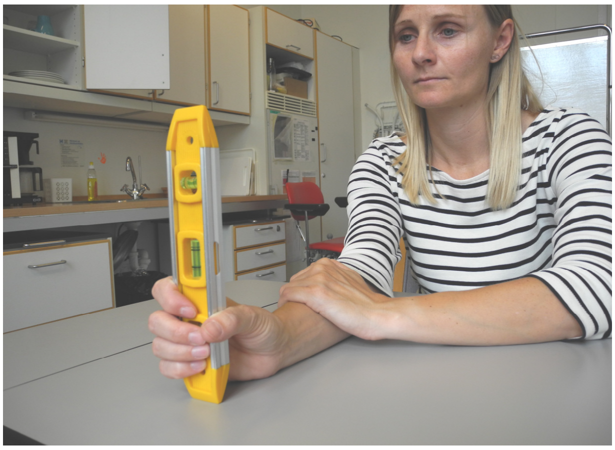

Exercise 17

- The patient pronates/supinates the paretic forearm while holding a bubble level that gives feedback on whether the movement goal is accomplished.

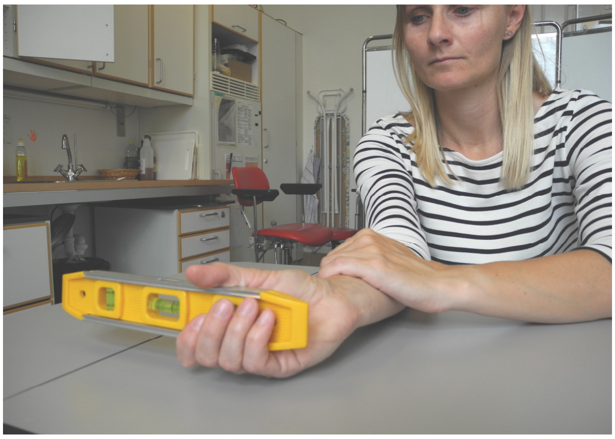

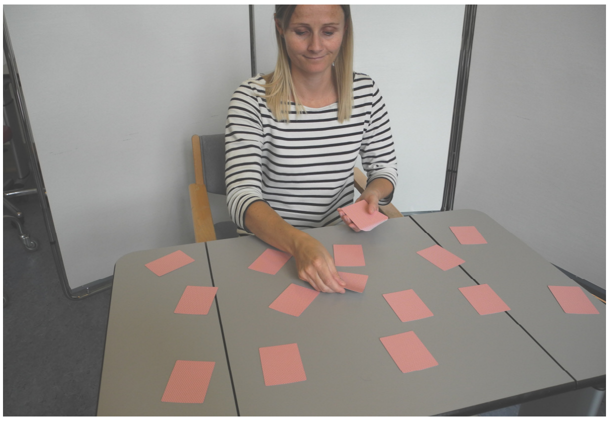

Exercise 18

- Pronation/supination.

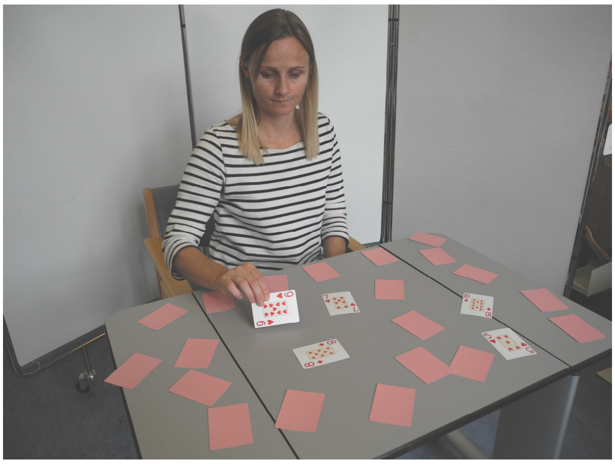

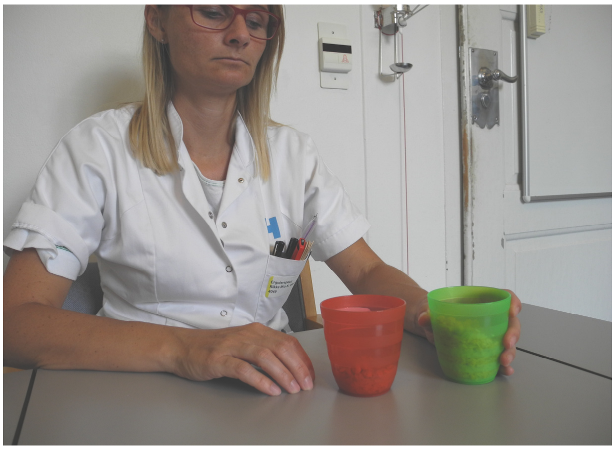

Exercise 19

- Pronation/supination.

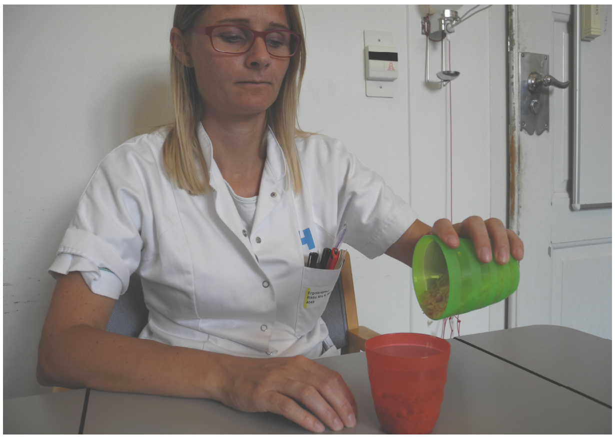

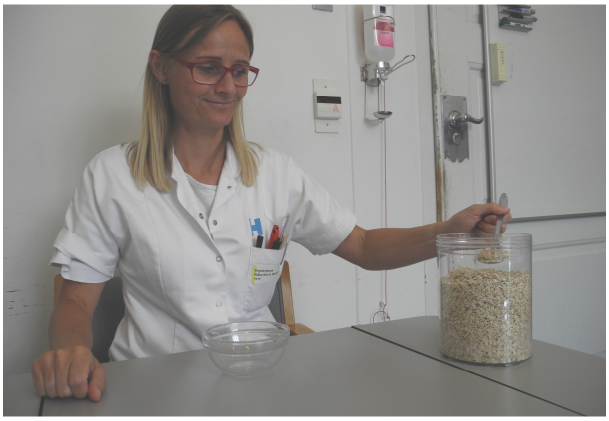

Exercise 20

- Pronation/supination, abduction/adduction of the shoulder.

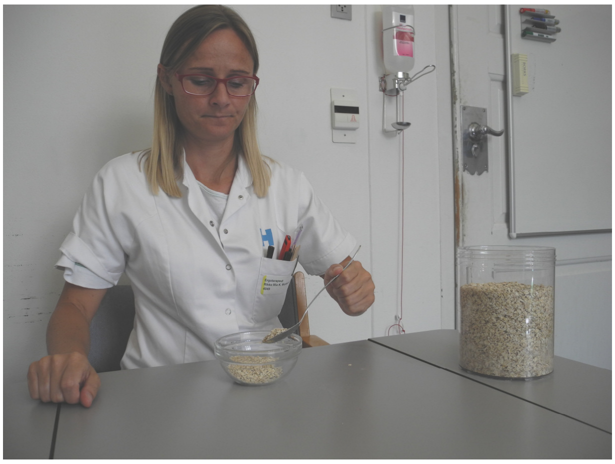

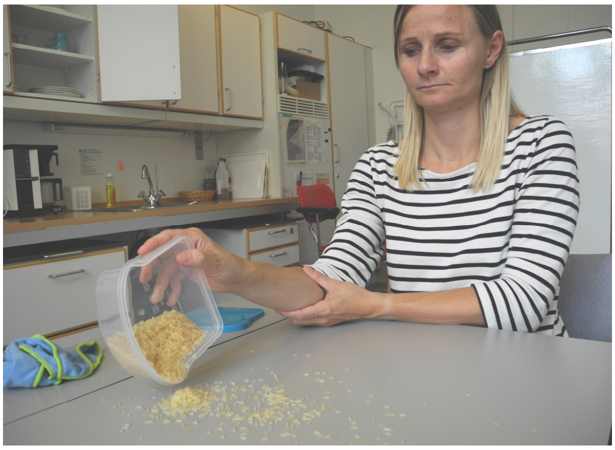

Exercise 21

- The patient spreads rice on the table and uses the paretic arm to sweep the rice in a box.

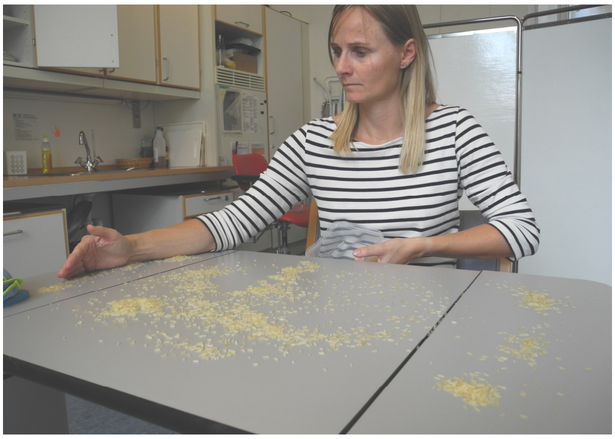

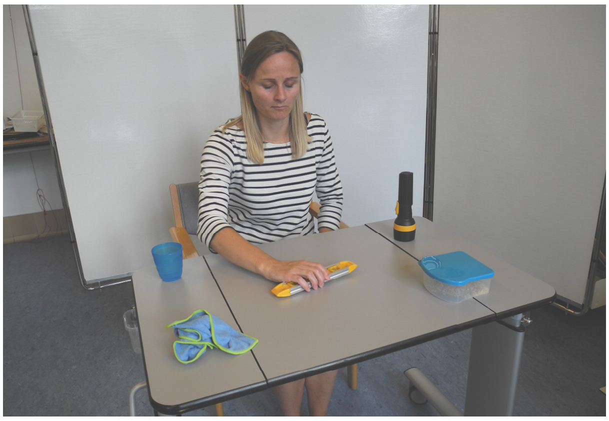

Exercise 22

- The patient places different objects on the table as shown in the picture.
- The patient grasps the objects and places them into a box on the floor.

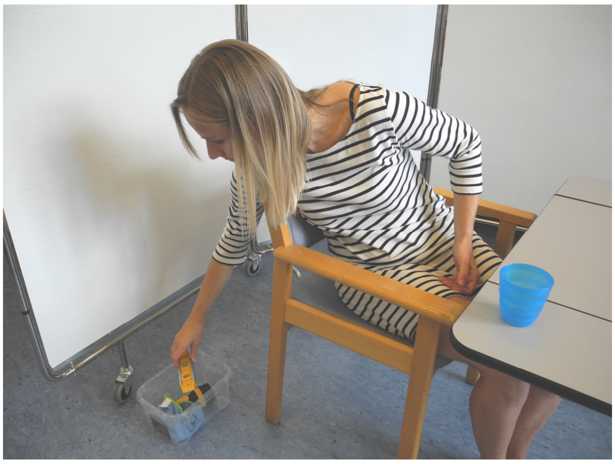

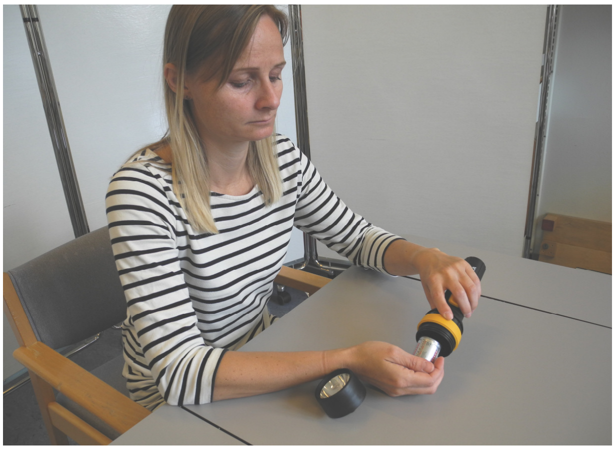

Exercise 23

- The patient holds the flashlight in her non-affected hand and changes the batteries with the paretic hand.

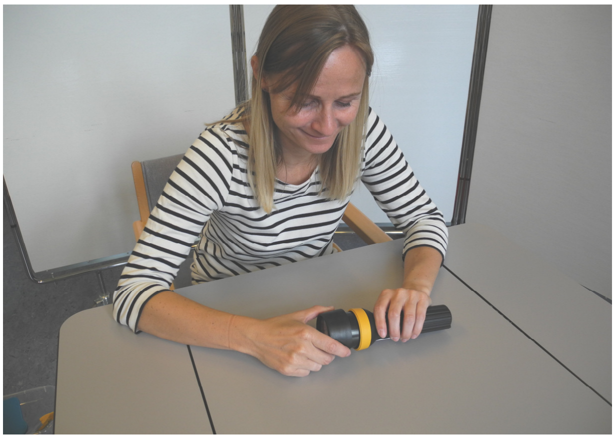

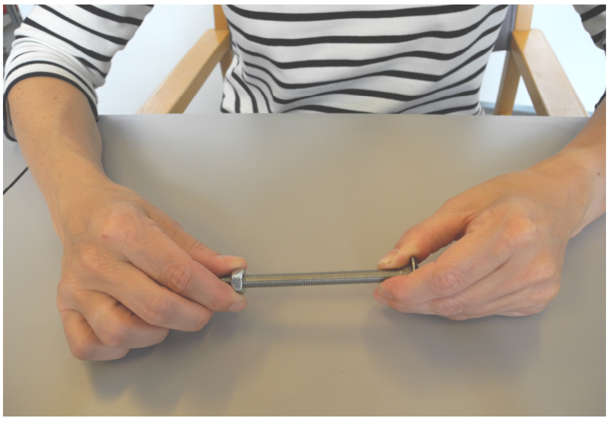

Exercise 24

- The patient has to assemble and disassemble nuts on the bolt.

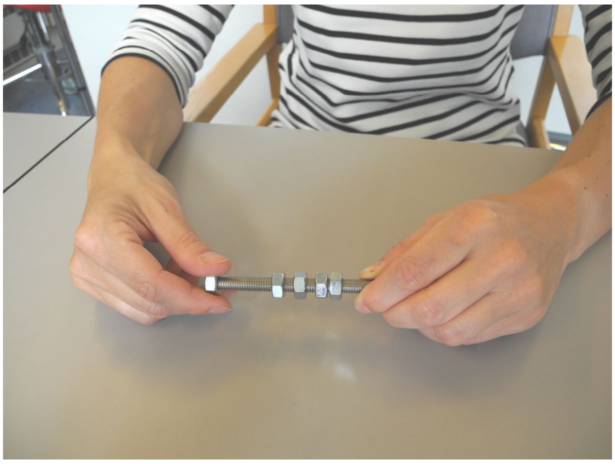

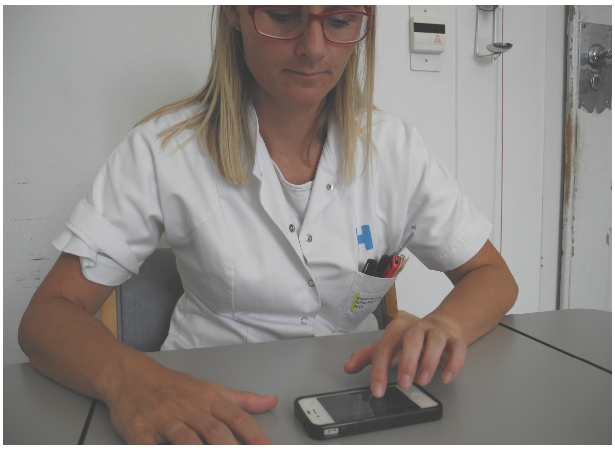

Exercise 25

- The patient writes text messages on a phone.

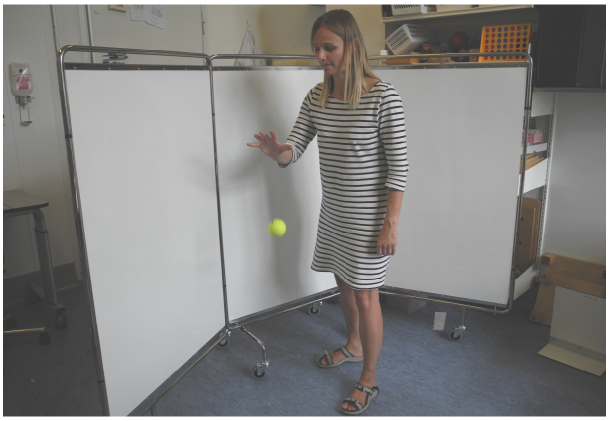

Exercise 26

- The patient throws a tennis ball against the floor and catches it with the paretic hand or both hands.

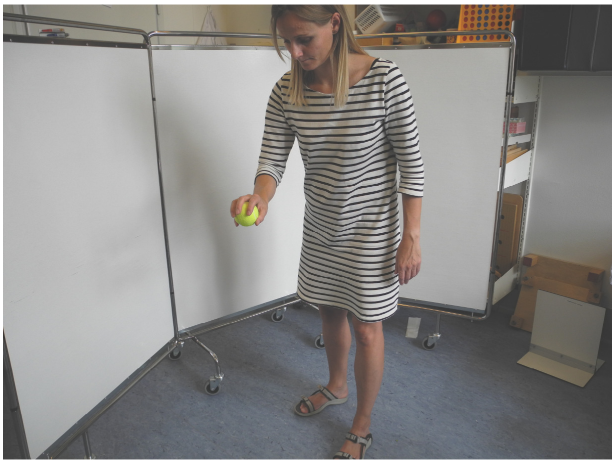

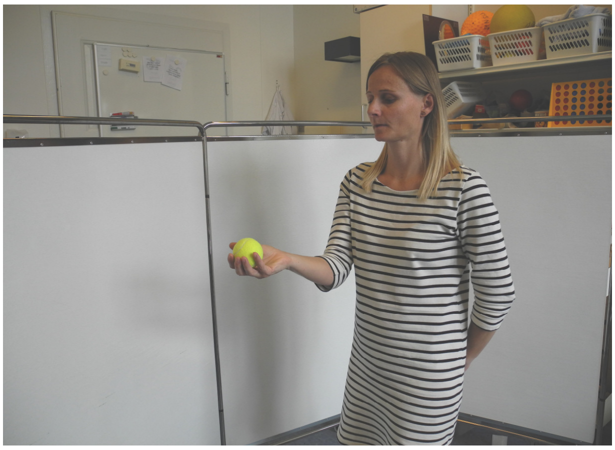

Exercise 27

- The patient throws a tennis ball up in the air and catches it with the paretic hand or both hands.

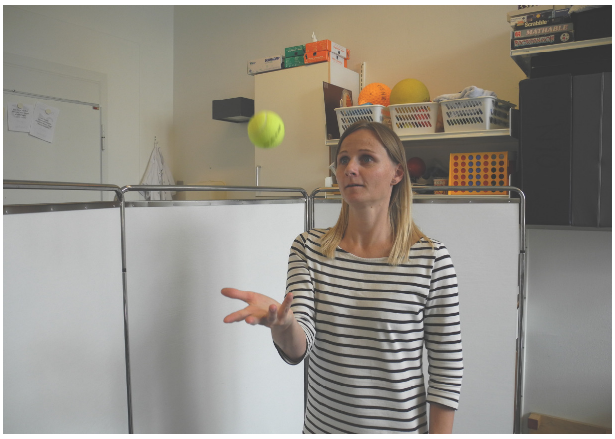

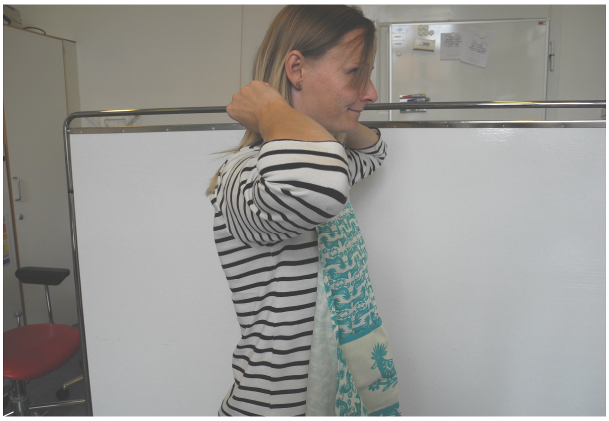

Exercise 28

- The patient uses both hands to put on an apron, ties it, and then takes it off.

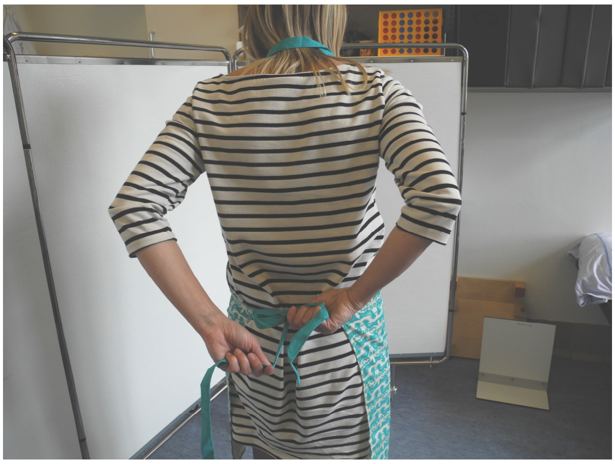

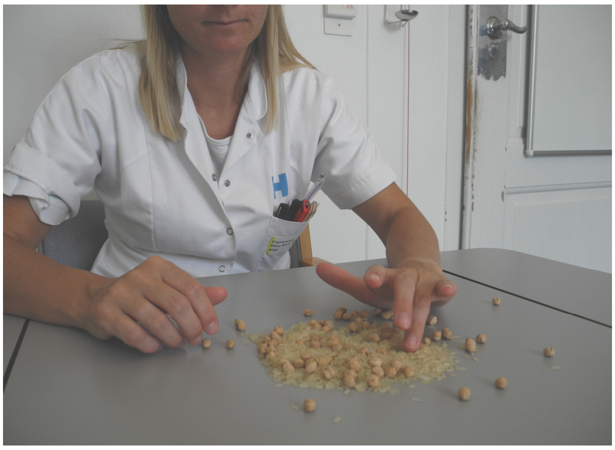

Exercise 29

- The patient spreads a mix of chickpeas and rice on the table. The patient uses each finger of the paretic hand to sort the chickpeas from the rice.

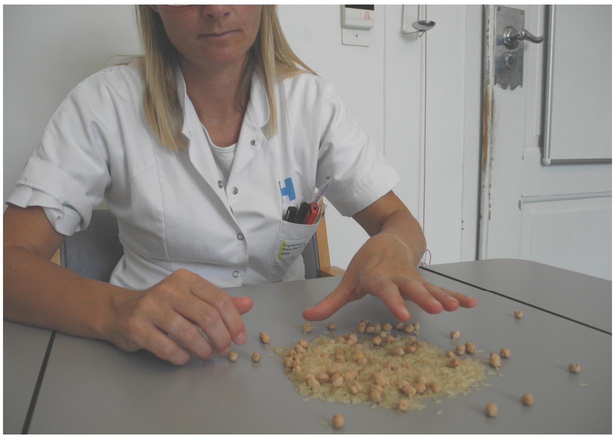

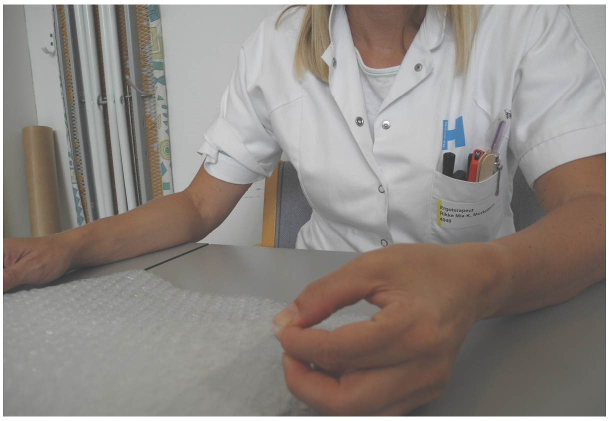

Exercise 30

- The patient uses the thumb and index finger of the paretic hand to brake bubble wrap.

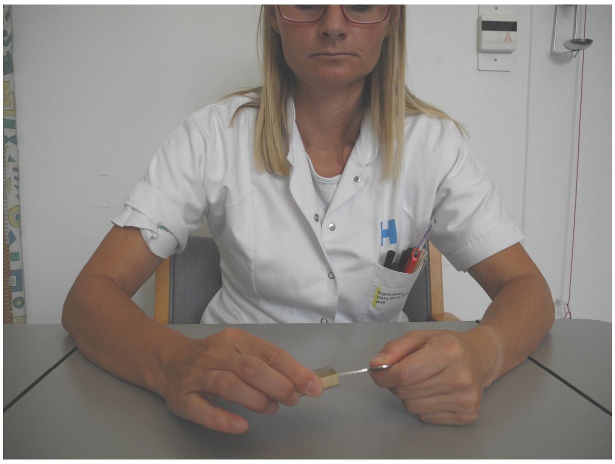

Exercise 31

- The patient holds the padlock with the non-affected hand. The patient uses the paretic hand to turn the key unlocking/ locking he padlock.

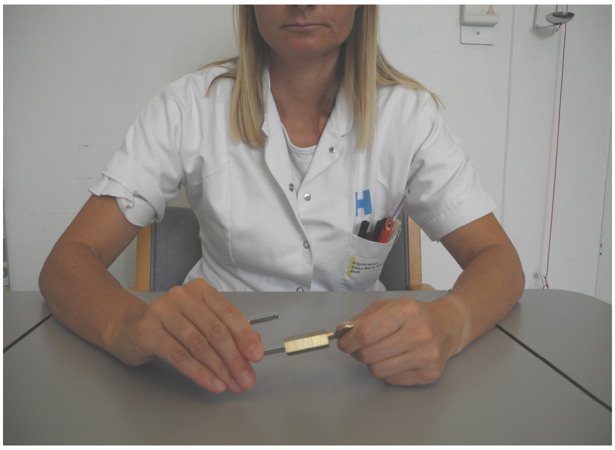

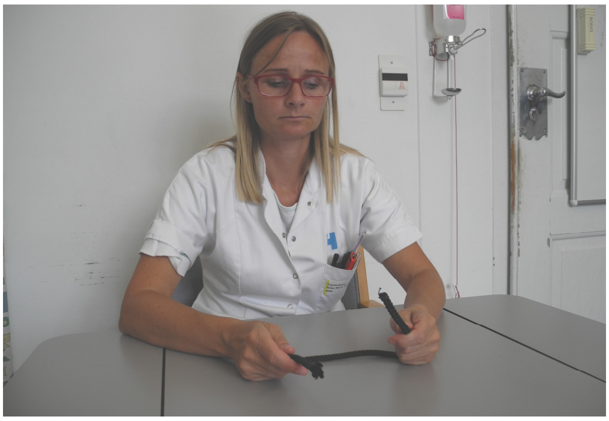

Exercise 32

- The patient uses both hands to tie a knot on a rope and then ties it up.

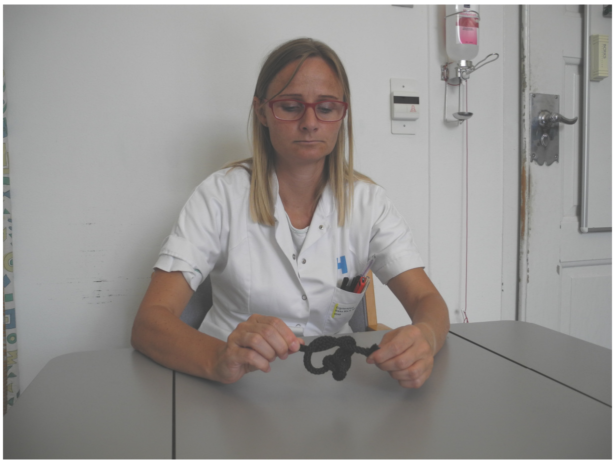

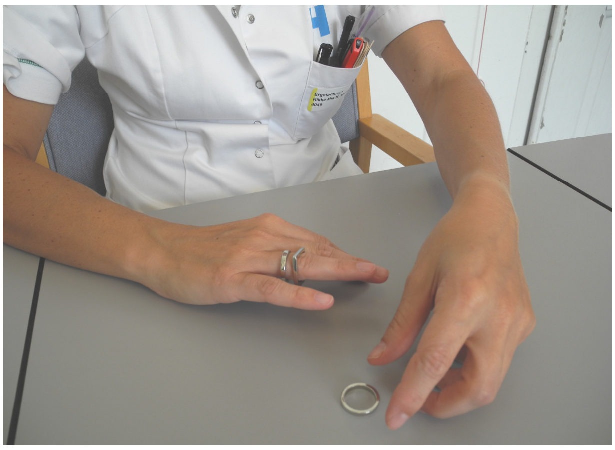

Exercise 33

- The patient takes a ring on and off her finger by using the paretic hand.

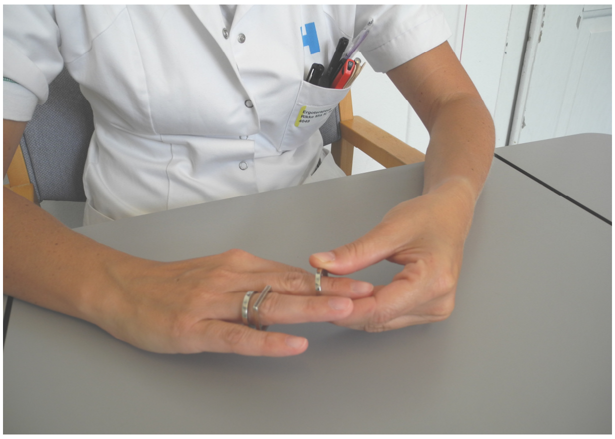

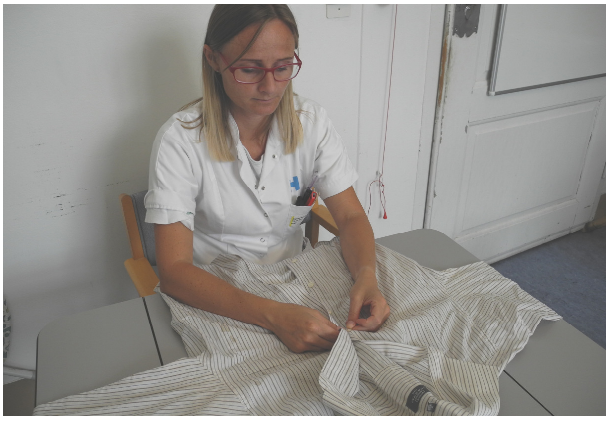

Exercise 34

- The patient buttons a shirt and then buttons it up.

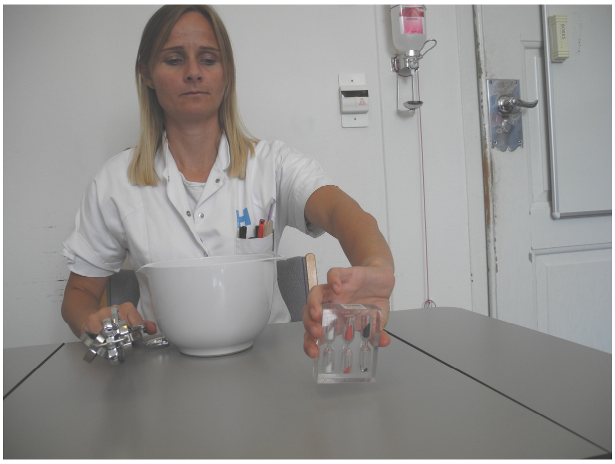

Exercise 35

- The patient holds a manual beater with the non-affected hand and activates it with the paretic hand for as long as it takes for the sand to run through a hourglass.

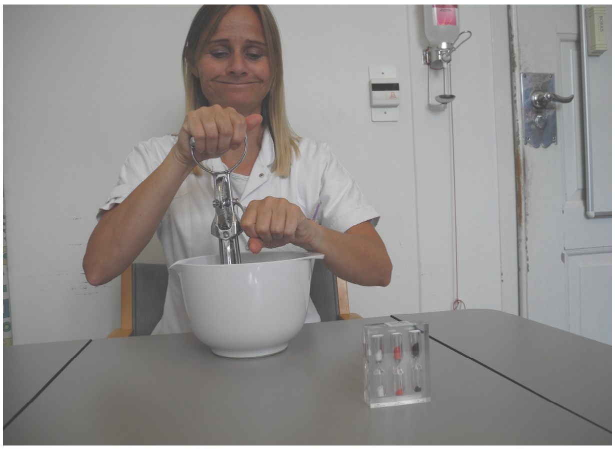

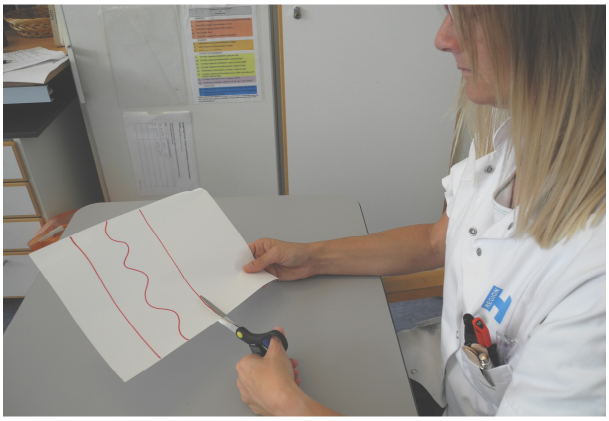

Exercise 36

- The patient cuts with a scissor.

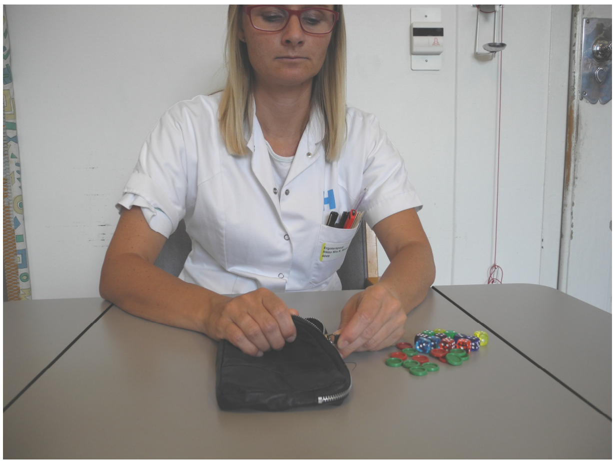

Exercise 37

- The patient places small objects in a purse.

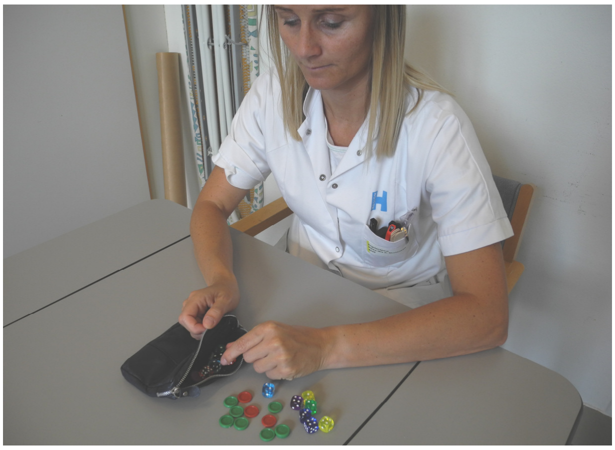

Exercise 38

- The patient plays solitaire.

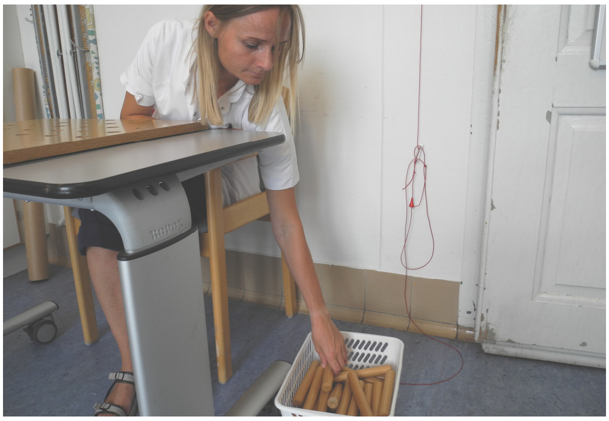

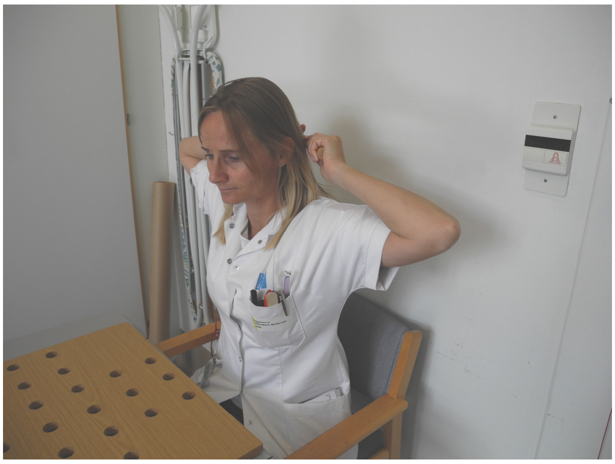

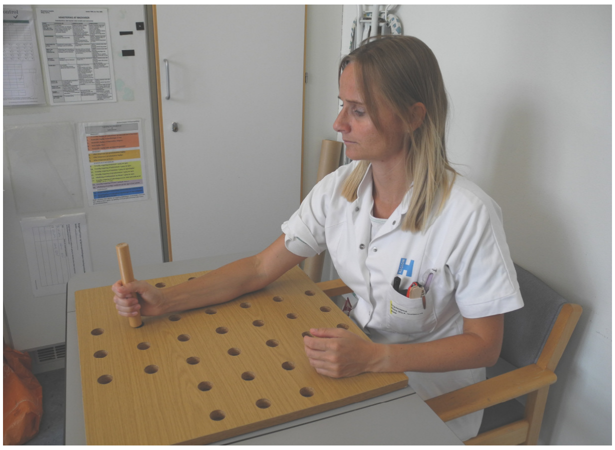

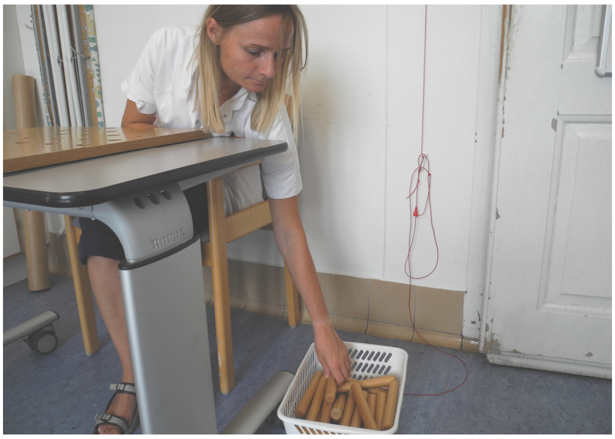

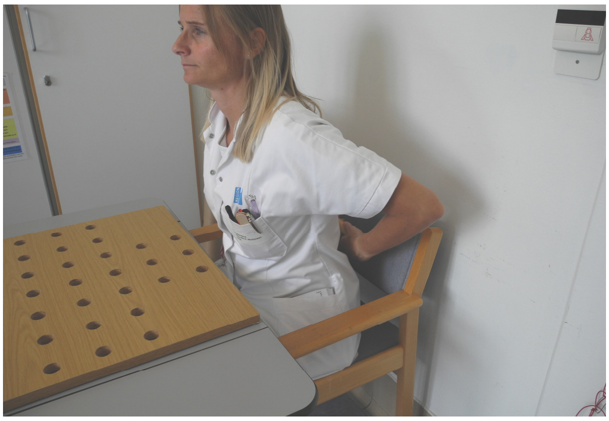

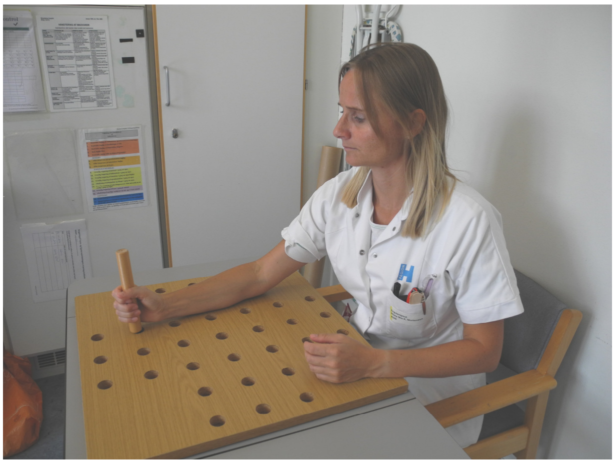

Exercise 38

- Continued.

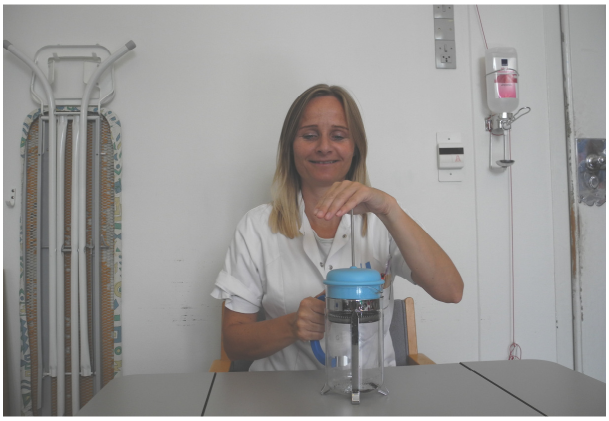

Exercise 39

- The patient brews coffee using a coffee press.

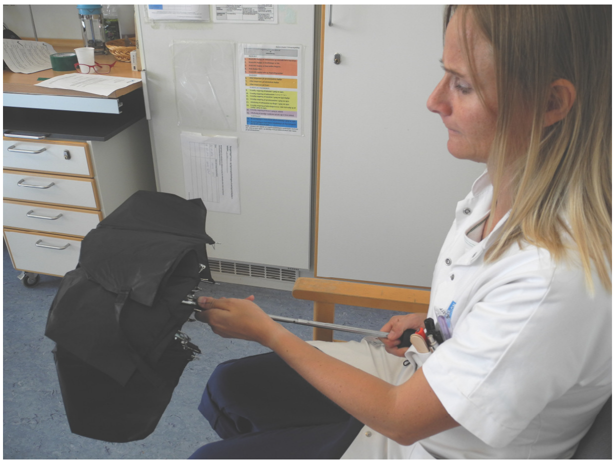

Exercise 40

- The patient folds an umbrella out.

Exercise 41

- Performance of other (parts of) ADL-tasks.
